# Applications of NIR Spectroscopy and Chemometrics for Food Authentication and Safety: Detection of Adulterants and Contaminants

**DOI:** 10.3390/molecules31173067

**Published:** 2026-08-31

**Authors:** Vanessa Pellicorio, Raffaella Colombo, Adele Papetti

**Affiliations:** 1 Department of Drug Sciences, University of Pavia, Viale Taramelli 12, 27100 Pavia, Italy; vanessa.pellicorio@unipv.it (V.P.); raffaella.colombo@unipv.it (R.C.); 2 Department of Drug Sciences & C.S.G.I., University of Pavia, Viale Taramelli 12, 27100 Pavia, Italy

**Keywords:** NIR spectroscopy, food fraud, adulteration, contamination, portable instruments, chemometrics

## Abstract

Adulteration and contamination represent an ever-growing global problem, due to the emergence of increasingly sophisticated illicit commercial practices involving high-value and widely consumed products. Traditional chromatographic methods, such as liquid chromatography coupled with mass spectrometry, offer high sensitivity but their application is often limited by long analysis time, complex sample preparation, and the use of non-eco-friendly organic solvents. In contrast, enzymatic and immunoassay-based methods are rapid and require minimal sample preparation, but their applicability may be restricted by antibody specificity and potential cross-reactivity. In this context, near-infrared (NIR) spectroscopy is a rapid, non-destructive technique that does not require the use of solvents and is becoming increasingly relevant in food analysis. This review provides an overview of the potential of this spectroscopic technique, coupled with chemometrics, for the detection of adulterants and contaminants in various matrices. After a brief summary of the principles on which it is based and the instruments that can be used, the chemometric processes useful for data interpretation have been discussed, as well as the main applications in liquid and solid food, including oils, milk, juices, spices, cereals, coffee, and dietary supplements. The analyzed case studies indicated that even very low levels (ppm) of adulterants and contaminants can be detected with high accuracy and sensitivity using models such as PLSR, SVM, RF, and CNNs and new and emerging devices such as portable ones.

## 1. Introduction

Food fraud is a global phenomenon that has gained increasing attention in recent years due to its significant impact on public health, consumer trust, and the global economy. According to the Global Food Fraud Index, the incidence of fraud showed marked fluctuations throughout 2025, with notable seasonal variations influenced by factors such as agricultural cycles, transportation dynamics, and supply chain vulnerabilities. These trends were consistent with data from FOODAKAI, an AI-based predictive platform, which reported an increase in fraud incidents during 2025, particularly affecting high-value products such as nuts, seeds, dried fruits, honey, syrups, and dairy products [1].

In Europe, food frauds have been continuously monitored through official notifications which documented a stable number of suspected cases, mainly concerning adulteration, counterfeiting, or contamination. The products most frequently subjected to deliberate adulteration included olive oil, eggs, alcoholic beverages, cocoa, tea, fish products, processed meats, fruit and vegetables, and especially food supplements, a rapidly growing sector that is often the victim of misleading labelling and non-compliant ingredients [2,3].

To protect consumers, there is already cooperation among established alert systems such as the Rapid Alert System for Food and Feed network which collects notifications regarding food, feed, food contact materials, and food supplements potentially hazardous to health, the Administrative Assistance and Cooperation network which reports administrative, documentary or commercial non-compliance, and the Food Fraud network which deals with reporting the most common cases of fraud and the patterns used to perpetrate it. These three systems provide a comprehensive image of the most common types of fraud, the patterns followed even at an administrative level, and the health risks that may arise from these illegal practices [2,3]. In 2026, the TraceMap platform was also introduced in Europe. It is an advanced system based on artificial intelligence that integrates data on trade flows, official controls, various alerts, and customs information. It enables the identification of suspicious events and assists national authorities in managing fraud, contamination, and various recalls [4].

The above-mentioned systems are all designed to maintain the concept of food integrity, which can be assured by focusing on four distinct issues: food safety, food defense, food quality, and food fraud. By acting on food safety, defense, and quality, there are certainly benefits in both economic and health terms, as action is taken before the product can reach the market and therefore pose a risk to public health. But, very often, the conceptual distinction between these issues is not entirely clear, although it is an important element in building systems that are effective for prevention and control throughout the agri-food supply chain. The most recent literature highlights how these issues share the final goal of safeguarding product integrity and consumer confidence but differ in terms of the nature of the risk and the strategies used to maintain integrity. In fact, food safety concerns the prevention of biological, chemical, or physical contamination, while food defense addresses intentional actions aimed at causing harm to public health or destabilizing the food system, such as product sabotage, extortion, or contamination driven by ideological motives. Food quality, on the other hand, aims to protect the quality of materials and ingredients used in the production of the final product [5,6]. Where it is not possible to address these three specific topics, it is necessary to prevent food fraud.

From the conceptual distinction among the above-mentioned issues, it is already clear that food frauds are intentional, as for adulteration, enrichment, and counterfeiting which are strictly deliberate. Differently, contamination, which can be physical, chemical, biological, allergenic or cross-contamination, depends both on the food matrix and on the stage of production at which it occurs, and can be accidental or often linked to human errors, negligence, or poor hygiene. Similarly, food alteration can be natural and spontaneous, but also intentional, depending on unsuitable and unsafe food storage [5,6,7,8].

Plant and cereal matrices can be contaminated by mycotoxins produced by fungi such as *Aspergillus*, *Penicillium*, and *Fusarium*, depending on humidity levels and storage conditions [9]. Differently, heavy metals such as lead, cadmium, arsenic, and mercury can originate from the soil, but also from the water used for irrigation or from environmental contamination, leading to the persistence and bioaccumulation of these substances [10]. Other contaminants are pesticides which may be mainly present in agricultural products due to treatments carried out prior to harvest, or due to cross-contamination or improper application [7,11]. Furthermore, contamination can also be caused by the packaging used for the product, which may degrade and release microplastics and nanoplastics, or other toxic chemical compounds, such as plasticizers or additives, mainly under mechanical stress, heat exposure, or prolonged contact with fatty foods [7,11,12,13,14].

The concept of food fraud involves all actions that are deliberately carried out and are often motivated by economic considerations. These can be divided into two main categories: commercial fraud and health fraud. The former causes economic harm to the consumer or the market and includes all behaviors that infringe on consumer rights without necessarily compromising their health. Typical examples include false claims regarding food origin, misleading labelling, the substitution of lower-value ingredients without posing a health risk, and the sale of products that do not conform to their declared characteristics. The aim is to gain an economic advantage by manipulating key information. Differently, health-related fraud is considered the most dangerous as it can pose a direct risk to consumer health. This includes, for example, the introduction of hazardous substances and/or manipulations and treatments that may cause biological, chemical, or physical harm. Very common examples include the addition of toxic substances such as melamine to milk to falsify its protein content or alterations that promote microbial proliferation. In any case, commercial and health frauds very often overlap, as certain unlawful practices originating as economic offences can very often lead to health risks [15,16,17]. Intentional food fraud is globally one of the main threats to food integrity and can be further divided into several categories, including adulteration, counterfeiting, document falsification, tampering with traceability, theft and illegal reintroduction, and unauthorized enhancement (Table 1). Specifically, adulteration is the deliberate addition of declared or unauthorized substances to increase the volume of the food or to reduce costs, as in the case of adding refined oils to extra virgin olive oil or cheap sugars to honey [18]. Dilution is often considered a subcategory of adulteration and consists of the addition of liquids or water to increase the volume of the product, a widespread phenomenon in the case of milk or fruit juices [19]. Enrichment, a historical fraud category now included in adulteration, is the artificial enhancement of a product’s sensory or qualitative characteristics using coloring, flavoring, or undeclared physical treatment to mask defects or simulate superior quality. One of the products most frequently subjected to this type of fraud is extra virgin olive oil, which is in part replaced with lower-grade seed oils that lighten the product and added with chlorophyll to restore the oil’s typical color [20]. Differently, substitution is the total or partial replacement of expensive ingredients with cheaper ones, a particularly widespread fraud in the meat and fish markets [21]. A practice widely diffused is counterfeiting which refers to the illegal reproduction of trademarks, packaging, or certification, whilst document falsification involves the intentional manipulation of labels, nutritional claims, or geographical origin. Other widespread practices are the manipulation of traceability, which is carried out by altering batches, expiry dates, or transport documents, and theft followed by illegal reintroduction on the market. Finally, unapproved enhancement involves the use of prohibited or undeclared substances to improve yield, color, or consistency, such as the addition of unauthorized additives [22].

From a scientific perspective, various analytical techniques are employed to detect adulterants and contaminants, such as enzymatic and chemical assays, calorimetric, spectroscopic, and separation-based techniques [23].

Colorimetric and enzymatic assays are often used for initial screening because they need short time and require minimal sample preparation. Immunoenzymatic methods such as Enzyme-Linked Immunosorbent Assay (ELISA) play a predominant role in detecting protein adulteration, allergens, antibiotic residues or specific contaminants, thanks to the high sensitivity of modern commercial kits, including fluorescent-based assays [24,25,26,27]. However, their applicability remains limited by antibody specificity and cannot reveal unexpected or non-targeted adulterants/contaminants, thus requiring complementary analytical techniques to complete the analyte identification.

Calorimetric techniques, such as differential scanning calorimetry (DSC), exploit the ability to assess the food thermal properties. Thanks to this property, they have been widely used for detecting adulteration in oil, butter, and dairy products, as these substances modify the food lipid composition, altering the crystallization and melting profiles [28,29]. However, although this technique is able to detect even minor structural changes, it has low chemical specificity and requires a comparative technique.

The most used techniques used for fraud prevention are the separative ones, in particular high-performance liquid chromatography (HPLC) and gas chromatography (GC), often coupled with mass spectrometry (MS). Liquid chromatography coupled with MS (LC-MS) is widely used for the analysis of mycotoxins, pesticides, or drugs [30,31,32], while GC coupled with MS (GC-MS) is used for the characterization of volatile analytes, persistent contaminants, and organic adulterants (Table 2) [33,34]. These techniques offer high specificity and sensitivity to unequivocally detect and quantify the substance of interest even when present in the food in very low amounts. However, even though these techniques are considered highly effective, they have significant limitations, such as very long analysis time, often complex sample preparation, the need for highly qualified user, and the use of solvents that are not particularly eco-friendly, resulting in increased costs for each analysis and danger to the environment.

This is why it is becoming increasingly crucial to have rapid and non-destructive detection techniques in cases of fraud. In this context, spectroscopic techniques such as Nuclear Magnetic Resonance (NMR), Raman, fluorescence, Mid-Infrared (MIR) and Near-Infrared (NIR)spectroscopy can be utilized.

These methods are particularly effective for the authentication of oils, honey, milk, spices, and plant-based products, as they are capable of acquiring highly selective and informative spectra, thanks also to the possibility of integrating chemometric models and machine learning algorithms [35].

The spectroscopic technique that is gaining the most prominence in the scientific community is NIR spectroscopy, thanks to its versatility and ability to provide rapid, non-destructive analysis without the use of solvents, and without the need for any sample preparation. In recent years, in fact, the use of this technology has grown exponentially. It is based on the absorption of radiation by the harmonic and combinatorial vibrations of C–H, O–H and N–H bonds, and this enables it to analyze even particularly complex matrices such as milk, oil, cereal, honey, meat, and vegetable products, without the need for sample preparation and with acquisition time of just a few seconds. Furthermore, it is possible to obtain multiple types of information from a single spectrum. For these reasons, it is widely used to identify adulterations such as the addition of water to milk, the blending of lower-value oils, the species substitution in fish products, the spices adulteration with dyes, the adulteration of honey and other applications.

Its usefulness is enhanced by combining it with qualitative, quantitative or classification chemometric methods, which allow authentic samples to be distinguished from adulterated ones even when spectral differences are minimal [36,37].

Another main advantage of this spectroscopy is the ability to miniaturize the method using portable instruments that enable checks on site, in warehouses, at raw material entry points, preventing significant adulteration or contamination even before the final product is placed on the market.

Benchtop and portable instruments have the same physics, but the latter incorporate compact optics, low-power LED or laser sources, and miniaturized detectors, enabling spectra to be acquired in a matter of seconds and anywhere. These instruments can be used to prevent numerous instances of fraud with an accuracy comparable to that obtained with laboratory instruments, provided that the models are built on representative and up-to-date datasets. The combination of portability, speed, and the use of digital platforms such as cloud, app, and machine learning makes them highly promising for real-time monitoring of food authenticity [38,39,40].

**Figure 1:**
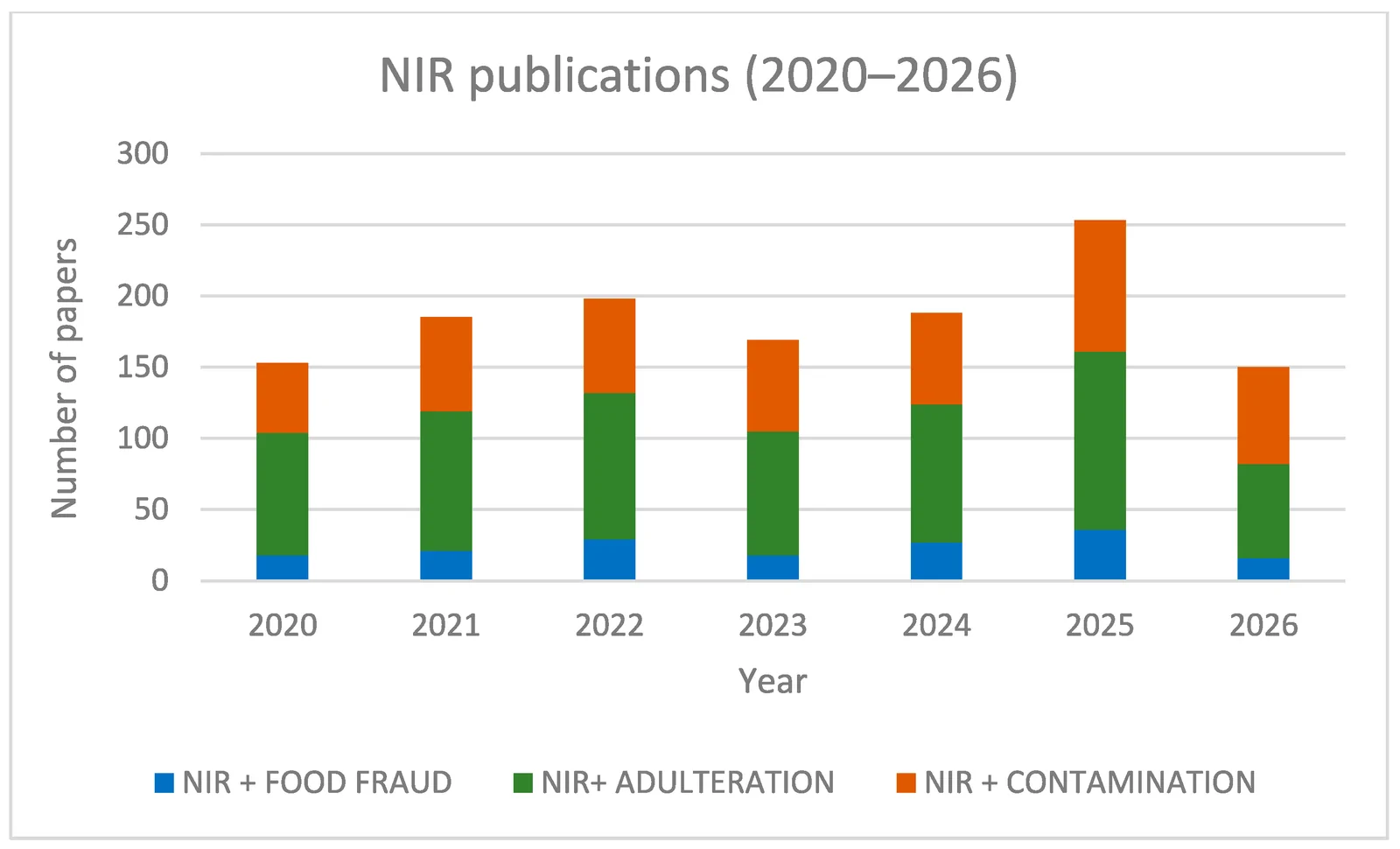
Annual distribution of NIR-based publications related to food fraud, adulteration, and contamination from 2020 to 2026.

The aim of this review was to provide a critical overview of the use of NIR spectroscopy as a rapid and non-destructive device that combined with chemometrics provides all the necessary tools for detecting various types of fraud throughout the agri-food supply chain. Most of the literature examined in this review has been published between 2020 and 2026. PubMed, Web of Science, and Google Scholar databases were used for the research using the keywords “NIR and adulteration”, “NIR and contamination”, and “NIR and food fraud”. Papers published before 2020 were also considered to contextualize the historical development of the technique and to illustrate how methodological approaches evolved over time. Figure 1 shows the distribution of published papers addressing NIR-based food fraud, adulteration, and contamination research in the last 7 years.

A comprehensive overview of the latest technological developments, both for benchtop and portable instruments, was provided to summarize all the advantages, critical issues, and future perspectives of a technique that could be a valid alternative to those currently in use.

## 2. NIR Instrumentation

NIR spectroscopy is an analytical technique based on the interaction between an electromagnetic radiation in the range of 780–2500 nm and the vibrational bonds (mainly C–H, O–H, N–H, and S–H) present in the molecules. Unlike the mid-infrared region, which is governed by fundamental vibrational transitions, the near-infrared region is dominated by overtones, i.e., higher harmonics, and by combinatorial bands. These transitions are less intense and more overlapping than the fundamental ones but are greatly affected by matrix structural or physico-chemical variations. The absorptions observed between 1200 and 2200 nm originate from overtone and combination bands of fundamental vibrational modes. The first overtone of C–H stretching typically appears around 1200–1300 nm, while the O–H first overtone is located near 1450 nm. The O–H combination band, arising from simultaneous stretching and bending vibrations, is centered around 1900–1950 nm, and N–H combination bands are commonly found in the 2050–2180 nm region.

**Figure 2:**
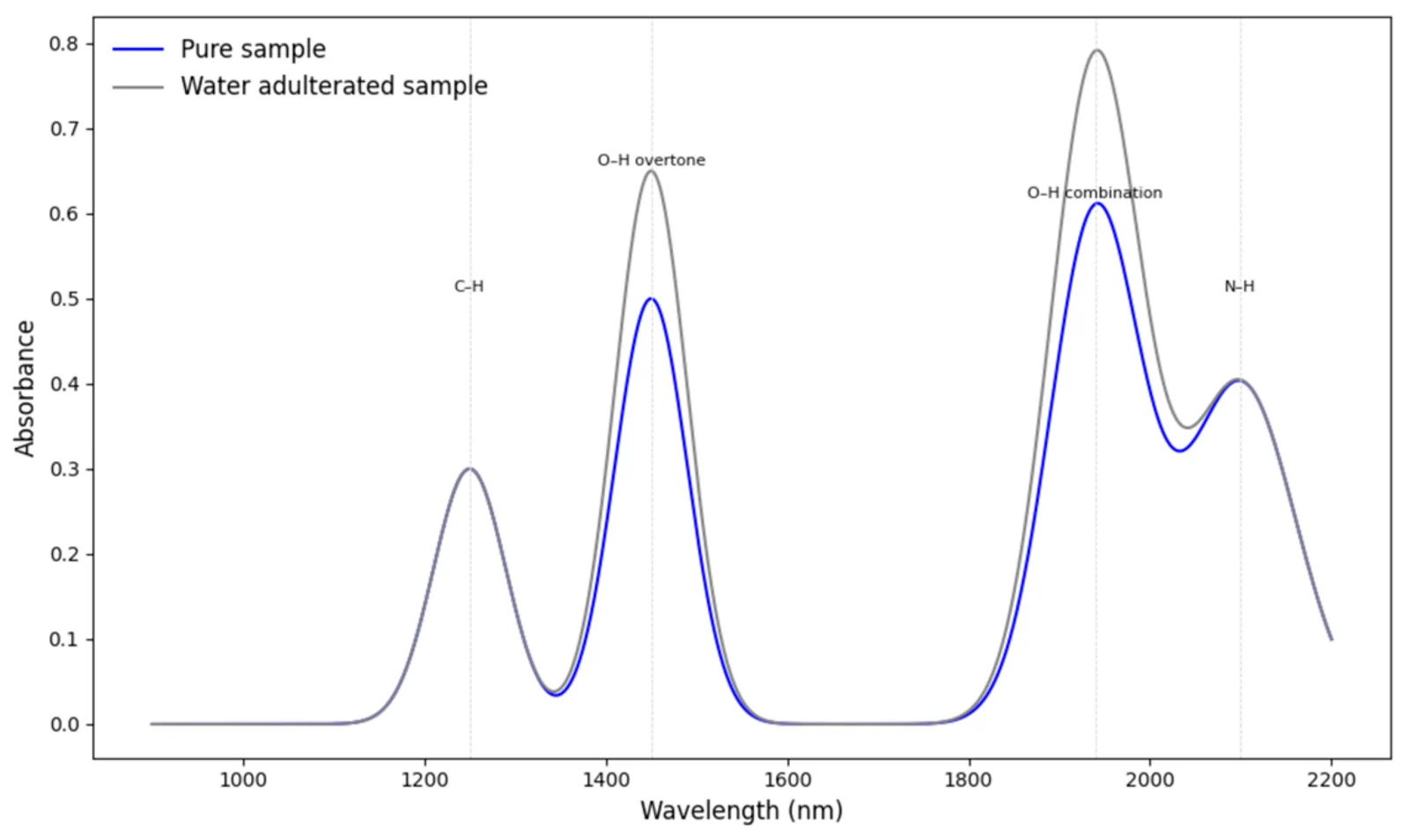
Representative NIR spectra of a pure sample (blue) and a sample adulterated with water (gray). The O–H overtone (≈1450 nm) and O–H combination band (≈1940 nm) show the expected increase in absorbance upon water addition, while the C–H and N–H regions remain essentially unchanged. This figure is a simulated example included to illustrate the typical spectral changes induced by water addition.

These spectral contributions are highly sensitive to changes in molecular composition, particularly to water content due to the strong O–H vibrational transitions. As an example, Figure 2 shows a comparison between the NIR spectra of a pure and an adulterated with water sample, demonstrating how NIR spectroscopy responds to adulteration involving water, providing a straightforward physical interpretation of the spectral variations typically observed in real samples.

Therefore, a NIR spectrum contains particularly complex bands, which make the technique multivariate.

NIR spectroscopy is historically based on IR spectroscopy and therefore on the absorption spectroscopy principles governed by Lambert-Beer’s law, which establishes the linear relationship between absorbance, concentration, and optical path length:

*A* = *ε*∙*c*∙*l* (1)

where *A* is the absorbance, *ε* (L·mol^−1^∙cm^−1^) the molar extinction coefficient, *c* (mol∙L^−1^) the concentration, and *l* the optical path length (cm).

Foods are heterogeneous and complex matrices, and the radiation undergoes multiple scattering due to the sample’s microstructure, specifically particle size, porosity, and physical state, which alter the linearity between absorption and concentration. Therefore, the spectrum observed at the end of the analysis is a combination of absorption and scattering, which is an informative component that must be processed using mathematical preprocessing and chemometric models to be intelligible [41].

NIR instrumentation encompasses a highly diverse range of optical configurations, light sources, and detectors; however, despite this variety, NIR instruments share three fundamental components: a radiation source, a wavelength-selection system, and a detector that is sensitive in the 780–2500 nm range (benchtop NIR) or between 900 and 1700 nm (portable and handheld NIR).

The most commonly used radiation sources are generally tungsten-halogen lamps, which are characterized by a continuous and stable spectrum, while portable devices use NIR Light Emitting Diodes (LEDs) or Micro-Electro-Mechanical Systems (MEMS) micro-sources to achieve miniaturization without compromising signal quality. LED sources used in simplified VIS/NIR systems typically provide narrow and well-defined emission bands, with peak-wavelength tolerances within ±5–10 nm, spectral bandwidths in the range of 20–40 nm, minimal peak shifts of 1–3 nm with drive current, radiant power between 55 and 100 mW, and repeatability values generally limited to 2–4% [42]. These characteristics are representative of high-power VIS/NIR emitters such as the ELJ series (Roithner Lasertechnik GmbH, Vienna, Austria), characterized by peak wavelengths of 630–850 nm, spectral bandwidths of 20–40 nm, and radiant power of 55–100 mW. Commercially available InGaAs-based infrared LEDs, such as the Hamamatsu L12771 series (Hamamatsu Photonics K.K., Hamamatsu City, Japan), emit around 1300 nm with a spectral half-width of approximately 90 nm and radiant flux values in the 2–4 mW range at 50 mA, making them suitable reference sources for moisture-related NIR applications [43]. GaAs-based emitters such as the OSRAM SFH 4775S (ams-OSRAM AG, Premstaetten, Austria), centered between 912 and 950 nm with a typical full-width at half-maximum of 37 nm and radiant flux values of 1000–1600 mW at IF = 1 A, exemplify the high-power, narrow-band sources available in the VIS/NIR region. Together, these InGaAs- and GaAs-based devices illustrate the breadth of spectral and radiometric properties accessible for modular LED-based VIS/NIR systems, supporting the flexibility required to tailor emission bands to specific analytical applications [44]. LED sources are very compact and energy-efficient, with a long operational lifespan (typically >30,000–50,000 h under standard operating conditions); very often, multiple LEDs with different wavelengths are combined. Conversely, MEMS sources integrate miniaturized optical and mechanical components directly into silicon chips, thereby achieving compactness and high performance. Miniaturized MEMS-based spectrometers typically operate in narrower spectral windows (e.g., 900–1700 nm), with spectral resolutions around 10–20 nm and signal-to-noise ratios of a few hundred to several thousand, depending on the architecture [45]. MEMS spectrometers frequently employed in compact analytical devices include systems such as the Texas Instruments DLP^®^ NIRscan Nano (Texas Instruments, Dallas, TX, USA) and the Si-Ware Systems NeoSpectra Micro (Si-Ware Systems, Heliopolis, Cairo, Egypt), which are widely integrated into portable platforms owing to their reduced footprint and suitability for low-power and on-site measurements [46,47]. Tungsten-halogen lamps are the most used in benchtop NIR spectrometers, as they produce a continuous spectrum covering uniformly the entire region 780–2500 nm. They are more stable than the sources used in portable devices because the tungsten filament is in an atmosphere containing halogen gases and reaches high temperatures that ensure greater stability and the acquisition of high-resolution spectra. However, these lamps consume more energy, generate heat, and require a longer warm-up time to reach operational stability.

The wavelength selection system consists of the optical components that isolate specific portions of the spectrum for signal acquisition. The simplest one is based on interference or narrowband filters which are static filters used in low-power systems. Linear Variable Filters (LVFs) and Linear Tunable Filters (LTFs) can be used as alternatives, especially in compact microspectrometers. LVFs are static interference filters with a variable bandwidth along a single dimension, while LTFs allow for tunable spectral selection through mechanical displacement of the filter [48].

In more advanced systems, spectral selection is performed by monochromators or diffraction gratings, which disperse radiation according to wavelength, allowing for continuous scanning of the spectrum. Non-dispersive benchtop spectrometers use interferometers to modulate the incident radiation and reconstruct the spectrum via Fourier Transformation. Differently, in new-generation portable instruments, the wavelength selection is performed by Digital Light Process/Digital Micromirror Devices (DLP/DMD) or Micro-Electro-Mechanical Systems (MEMS) which have the only purpose of selecting the wavelengths that will reach the detector. These architectures typically achieve spectral resolutions between 10 and 25 nm and enable rapid scanning without moving parts [45].

The most common detectors, named InGaAs (Indium–Gallium–Arsenide), are highly sensitive, have low noise, and offer fast response time. For these reasons, they are widely used in both portable instruments and benchtop spectrometers. The typical spectral window within which they operate is between 900 and 1700 nm, extendable to 2500 nm in extended-range versions. Other usable detectors are based on lead sulfide (PbS) and lead selenide (PbSe), sensitive up to approximately 2500 and 3000 nm, respectively, but characterized by significant noise and lower thermal stability. Some instruments use linear or two-dimensional InGaAs arrays, which allow the entire spectrum to be acquired without mechanical scanning, thereby improving speed and reproducibility. In portable devices, however, compact detectors, optimized for low power consumption, are used [49,50].

The nature of the information obtained from the spectrum largely depends on the measurement geometry and, consequently, on the instrument configuration which allows the radiation to interact with the sample. In food matrices, which are highly heterogeneous and exhibit significant scattering, it is crucial to properly set this parameter to obtain a sufficiently robust predictive model.

Diffuse reflectance is the configuration generally used for solids, powders, flours, cereals, and opaque products; in this case, the radiation penetrates the sample, undergoes multiple scattering events, and is then re-emitted toward the detector. The resulting spectrum will be dominated by scattering and volumetric interactions. Differently, transmittance is used for liquid, semi-liquid, or thin solid samples, as the radiation can pass through the sample along the defined optical path. In this case, the absorption component is more pronounced than the reflectance, given the greater linearity of the signal [49].

For turbid samples, emulsions, and matrices in which even transmittance would be too attenuated, transflectance is used. In this case, the radiation passes through the sample, is reflected by a mirror placed on the opposite side and returns to the detector [51]. Therefore, the measurement geometry is generally selected based on the matrix, the physical state of the sample, and the analytical goal.

### 2.1. Benchtop NIR

Benchtop spectrometers are the most stable instruments in near-infrared spectroscopy and include dispersive, Fourier-Transform Near-Infrared (FT-NIR), and array-based instruments.

Dispersive instruments are the most traditional and rely on the angular separation of wavelengths via a diffraction grating; specifically, the source radiation is collimated, dispersed, and refocused through a monochromator. The spectrum is sequentially acquired through the mechanical rotation of the grating.

The most common architecture in benchtop dispersive instruments is the Czerny-Turner configuration, in which light enters through a slit, is collimated by a concave mirror, dispersed by a diffraction grating, and refocused by a second concave mirror toward the detector [52].

FT-NIR instruments use a Michelson interferometer, which modulates the radiation to generate an interferogram to which the Fourier transform is applied, yielding the spectrum. This type of architecture is characterized by two main advantages: the multiplex advantage (or Fellgett advantage) which allows for the simultaneous acquisition of all wavelengths, and the throughput advantage (or Jacquinot advantage) which maximizes the amount of radiation reaching the detector due to the absence of narrow slits [52,53]. Commercial FT-NIR instruments (e.g., Bruker MPA II) achieve analytical accuracy around 0.03% for fat and protein and repeatability below 0.01%, thanks to high-stability tungsten-halogen sources and interferometric architectures. The large pathlength (1 mm) enables the analysis of viscous liquids, while in-line FT-NIR systems provide moisture accuracy of 0.15%, comparable to Karl–Fischer titration. The RockSolid™ cube-corner interferometer ensures long-term wavelength stability and robustness against vibrations and thermal drift, supporting reliable calibration transfer and high spectral quality [54].

A third category consists of array-based instruments, which combine a fixed diffraction grating with linear or two-dimensional arrays of detectors, typically InGaAs. Each element of the array receives a portion of the spectrum and all wavelengths are simultaneously acquired without mechanical scanning. This architecture avoids moving parts, thereby increasing the acquisition speed.

All the above-mentioned benchtop NIR instruments use high-stability sources, such as tungsten-halogen lamps and high-performance detectors such as InGaAs.

In recent years, Hyperspectral Imaging (HSI) has been used coupled with NIR spectroscopy, primarily for complex matrices. This technique simultaneously combines spectral and spatial information, generating the so-called hyperspectral cube, in which each pixel contains a complete spectrum. Hundreds of contiguous spectral bands are acquired and each band captures a narrow portion of the spectrum, allowing for the detection of subtle variations associated with functional groups or physical or chemical states [55].

### 2.2. Portable and Handheld NIR

In recent years, portable and handheld NIR instruments have become increasingly important because of their simple and quick on-site use. The miniaturization of these instruments has been made possible by the introduction of new spectral selection technologies, which replace the traditional benchtop methods, i.e., gratings and interferometers, that are too bulky and sensitive to vibrations to be incorporated into these smaller systems.

In fact, portable devices can incorporate an Acousto-Optic Tunable Filter (AOTF) which uses a birefringent crystal through which an acoustic wave passes. By selecting the wave on the device, it is possible to selectively diffract only the desired wavelength. Thanks to this technology, very fast scans can be acquired. Another solution adopted in portable systems consists of MEMS microspectrometers, which are typically based on Fabry–Pérot microinterferometers. In this case, the distance between the mirrors in the cavity is electrostatically modulated, allowing for the tunable selection of very narrow spectral bands. MEMS-based Fabry–Pérot and DLP/DMD architectures typically operate in the 900–1700 nm region with spectral resolution between 10 and 25 nm and SNR values ranging from 200 to 800, depending on the specific design [45]. This technology reduces the power consumption, as well as the technology based on DLP/DMD systems, in which light is dispersed by a miniaturized grating and then modulated by micromirrors digitally tilting to reflect specific portions of the spectrum toward the detector [48,56].

**Figure 3:**
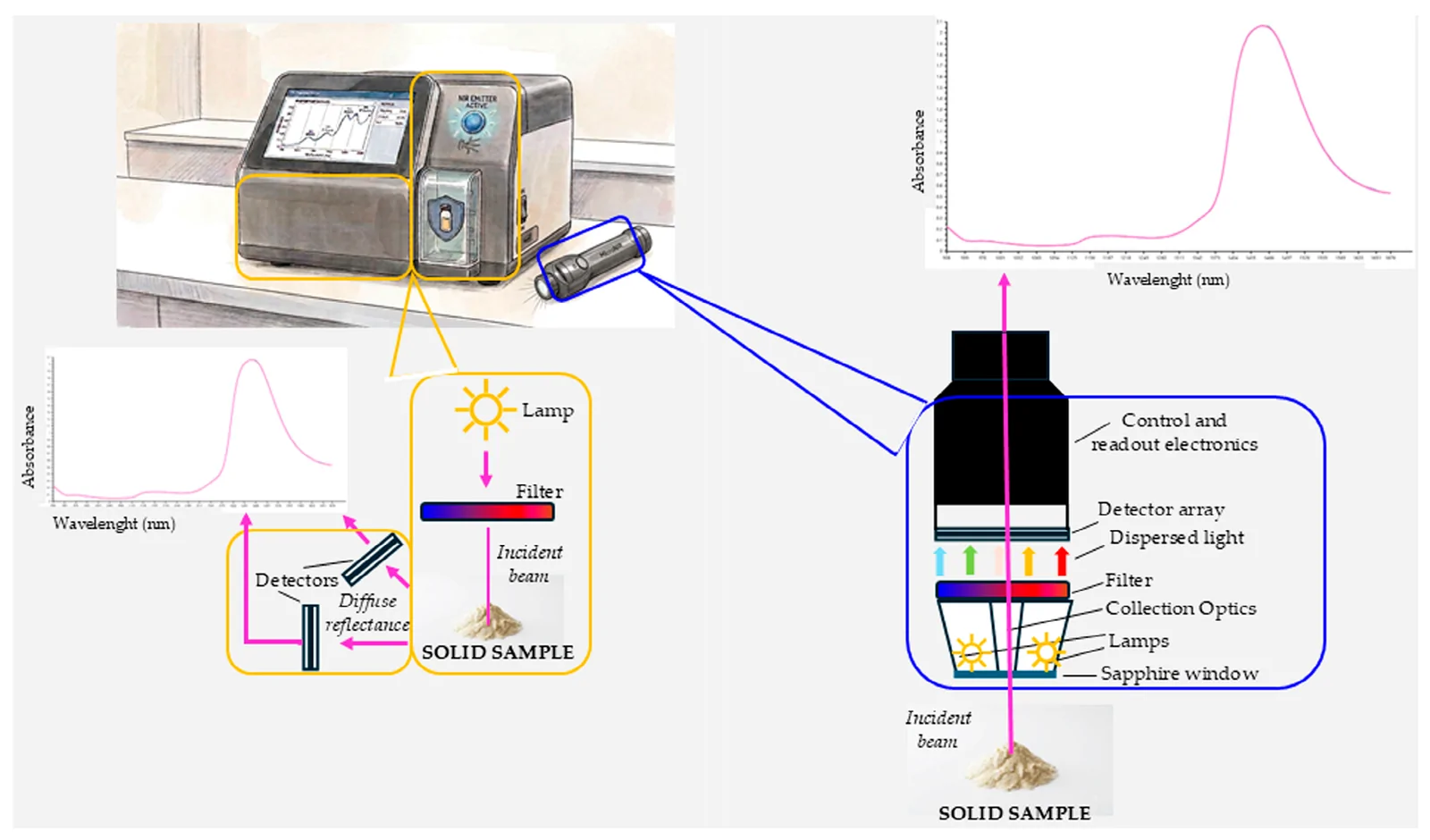
Schematic representations of NIR equipment (benchtop and portable instruments) and their configurations. The illustration of the two NIR instruments (benchtop-yellow line and portable-blue line) in the part at the top left has been generated using Google Gemini.

A schematic representation of benchtop and portable NIR equipment is reported in Figure 3.

## 3. Chemometrics and Data Processing

NIR spectroscopy generates highly complex data, with broad bands that overlap or are influenced by various factors. For this reason, chemometrics is mandatory to extract meaningful information from the spectra. In fact, a raw NIR spectrum incorporates contributions deriving from the sample’s chemical composition, scattering effects, variations in the optical path, instrumental noise, temporal drift, and differences in measurement geometry. Therefore, chemometrics is involved in three key phases: in the preprocessing step to remove variability, in the calibration step to build a model and establish quantitative or qualitative relationships between the spectra and their properties, and finally, in the validation to verify the model’s robustness and ensure it is both usable and efficient [57]. These three phases will be discussed below, and Table 3 reports a summary of a typical NIR analytical workflow.

### 3.1. Preprocessing Step

Preprocessing is the stage in which all non-chemical variations that may be present in the spectrum are corrected and eliminated; specifically, this includes contributions from physical and instrumental effects that can distort the spectrum, such as additive and multiplicative scattering, baseline shifts, noise, instrumental drift, or other physical differences. Without preprocessing, the modeling would be unstable and misleading.

There are various treatments that can be performed and in a data matrix they can be applied both to the rows (row pretreatment), i.e., to each individual spectrum, and to the columns (column pretreatment) by treating wavelengths as variables [49]. They are of fundamental importance because most of the unwanted variability in NIR spectra does not arise from chemical differences between samples, but from physical effects (e.g., scattering, baseline drift, noise) that distort the shape of each individual spectrum. Row preprocessing techniques correct these intra-spectral distortions, ensuring that the spectral information reflects the underlying chemistry.

Several treatments can be applied to the row (Table S1): Standard Normalized Variate (SNV) normalizes each spectrum relative to itself by subtracting its mean and dividing by its standard deviation. This method corrects additive and multiplicative scattering, slopes, and overall differences, thereby making the spectra comparable to one another. The only limitation of this preprocessing is that it does not correct non-linear effects, and if the standard deviation is small, it may amplify the noise. Differently, Multiplicative Scatter Correction (MSC) uses a reference spectrum, typically the point-wise mean of all spectra in the dataset, to correct differences in slope, offset, and multiplicative scattering. This aligns the spectra to a common reference and reduces physical variability. Detrending removes low-frequency trends in the individual spectra by fitting a local polynomial. It corrects for sloping baselines, instrumental drift, and temperature variation. The most important row-based preprocessing techniques are the Savitzky–Golay (SG) smoothing and derivatives. These include the first derivative, which removes a constant baseline, emphasizes changes in slope, and highlights broad bands, thus eliminating additive contributions and highlighting rapid signal variations, or the second derivative, which removes a linear baseline, separates overlapping bands, and increases apparent resolution, making hidden bands visible. Row-based pre-processing methods directly act on the optical distortions that arise during NIR acquisition. In diffuse-reflectance measurements, photons undergo multiple scattering events caused by particle size, surface roughness, refractive-index mismatches, and heterogeneity in packing density. These phenomena generate additive and multiplicative effects, baseline tilts, and curvature that mask the true absorbance associated with O–H, C–H, and N–H overtone, and combination bands. SNV and MSC correct these distortions by normalizing the intensity variations produced by scattering: SNV removes sample-dependent scaling and offsets, while MSC aligns each spectrum to a reference, compensating for differences in optical path length and scattering efficiency.

Detrending addresses low-frequency components arising from instrumental drift, temperature fluctuations, and gradual changes in scattering as the matrix becomes more or less compact. These slow variations are unrelated to molecular vibrations and therefore have to be removed to isolate chemically meaningful information.

Savitzky–Golay smoothing and derivatives operate on the frequency content of the spectrum. Smoothing reduces high-frequency noise originating from detector electronics or photon shot noise, while derivatives suppress low-frequency components associated with scattering and baseline drift. Since vibrational transitions produce sharper and higher-frequency features than scattering, derivative filters enhance chemically relevant bands while attenuating non-specific optical contributions. This effect is particularly important in heterogeneous food matrices, where particle size distribution and microstructure strongly influence the scattering profile. By reducing these matrix-dependent distortions, derivatives improve the visibility of overtone and combination bands and increase the chemical interpretability of the spectra [58,59,60].

Row preprocessing is therefore a fundamental step for correcting the physics and shape of the spectra (Table S1).

Column preprocessing steps prepare the data for modeling and include mean centering, autoscaling, and range scaling as the most important approaches.

Mean centering subtracts the mean of each variable (wavelength) calculated across all samples. This brings the mean of each variable to zero and facilitates the construction of linear models. For models such as Principal Component Analysis (PCA) and Partial Least Squares (PLS), this step is essential to avoid incorrect PCA identification of the direction of maximum variance and to prevent PLS from failing to extract the correct latent components. Autoscaling, also known as standardization, consists of dividing each variable by its standard deviation calculated across all samples. This ensures that all variables are comparable and prevents high-intensity spectral regions from dominating the model. It is used when variables have very different variances and when scale-sensitive methods are employed. The limitation of standardization in NIR applications is that it can amplify noise in less informative regions. In range scaling, each variable is scaled to a defined range so that the scale of the variables is standardized [58,59,60].

Applying these preprocessing steps (which affect the structure of the dataset) is important for stabilizing the modeling process, ensuring that variables are comparable, and enabling the models to properly function (Table S2).

Very often, it is necessary to apply a combination of column and row preprocessing techniques to the dataset to simultaneously correct both physical and static effects, thereby addressing the complexity of the signal more comprehensively. Generally, the workflow firstly involves the application of the row-based preprocessing, as it corrects the physical aspects of the individual spectrum. Next, column-based preprocessing is applied to stabilize the statistical structure of the dataset and prepare the data for multivariate modeling. This is a very important consideration, as reversing the order can alter the distribution of the variables and prevent the capture of the correct chemical variability.

The most used mixed preprocessing methods generally include SNV followed by mean centering, which is useful for correcting scattering and statistically centering the variables; MSC followed by SG derivative, which is particularly useful when both intensity differences and baseline shifts need to be corrected; or derivative and autoscaling, often used in classification when it is necessary to emphasize small differences among the samples.

In complex matrices such as heterogeneous food products, mixed pretreatments are typically used to obtain more robust models, reduce sensitivity to uncontrolled variations, and improve predictive power. However, the use of mixed preprocessing techniques requires caution, as improper use could alter the dataset’s output. Therefore, it is essential to evaluate the overall effect of preprocessing through exploratory analysis, such as PCA, and through validation, to ensure that the selected combination actually improves sample separation and correlation with the variables of interest [61,62].

### 3.2. Calibration

Calibration is the mathematical process performed to establish a quantitative relationship between spectra and the chemical–physical properties of interest.

In chemometrics, it is the process of learning a predictive function:

(2) $$\hat{y}=f(x)$$

where x is the sample spectrum and $\hat{y}$ is a reference value for the variables obtained through a primary analytical method.

Very often, before building models, variable selection strategies (also known as feature selection or feature extraction) are applied to improve the interpretability of the models and make them more robust. Among these, Variable Importance in Projection (VIP) represents one of the most widely used tools which quantifies the contribution of each variable, allowing the distinction between the most informative and redundant or noisy spectral bands. It is associated with PLS model, and it is not suitable for non-linear relationships. Differently, Net Analyte Signal (NAS) quantifies the portion of the signal attributable to the substance of interest, separating it from the component shared with other components in the matrix. It is effective for complex patterns, but there is an important risk of overfitting without proper validation.

By adopting the Competitive Adaptive Reweighted Sampling (CARS) technique, variables are selected through a process in which the least informative variables are eliminated, while the most relevant ones are retained and re-evaluated. It uses Monte Carlo sampling and PLS regression coefficients, and it is effective for high-dimensional spectra but unstable with small datasets. These three methods are the most widely used, but complementary approaches such as Genetic Algorithms (GA), (Iterative) Successive Projections Algorithm ((i)SPA), Uninformative Variable Elimination (UVE), Interval-PLS (iPLS), and Backward Interval-PLS (biPLS) can be used. These methods allow the variable space to be explored according to different logics, i.e., minimization of collinearity (as for SPA), elimination of uninformative variables or selection of contiguous spectral intervals (as for UVE), split the spectra in intervals and remove the uninformative variables (as for iPLS/biPLS), and evaluation of the regression coefficient stability and removal of less informative variables (as for UVE).

Variable-selection algorithms effectively operate in NIR spectroscopy because they target spectral regions where the underlying physicochemical phenomena generate measurable and reproducible variations. NIR spectra are dominated by broad, overlapping overtone and combination bands of O–H, C–H, and N–H groups, whose intensities and shapes are strongly influenced by molecular environment, hydrogen bond, conformational flexibility, and matrix-dependent scattering. As a result, many wavelengths carry redundant or noise-dominated information, while only specific regions reflect genuine chemical differences. CARS exploits this physical structure by repeatedly sampling subsets of wavelengths and retaining those characterized by the most stable and intense regression coefficients. During Monte-Carlo sampling, variables associated with weak or highly overlapped vibrational modes are progressively discarded, whereas regions containing sharper curvature or stronger absorbance, typically linked to fundamental O–H, C–H, or N–H interactions, are preserved. This makes CARS highly effective for high-dimensional spectra, although its reliance on repeated resampling can lead to instability in small datasets where spectral variance is limited.

Complementary algorithms explore the spectral space according to different physicochemical logics. SPA and iSPA minimize collinearity by selecting wavelengths that respond independently to molecular vibrations, thereby reducing the influence of scattering-induced correlations. UVE evaluates the stability of regression coefficients across perturbations, eliminating variables dominated by baseline drift, instrumental noise, or matrix-dependent scattering. Interval-PLS and biPLS exploit the fact that overtone and combination bands occur in contiguous spectral regions: by splitting the spectrum into intervals, they isolate segments where absorbance changes arise from specific functional groups, removing intervals dominated by noise or non-chemical variability.

Ultimately, the spectral regions selected by VIP, NAS, CARS, GA, or interval-based methods correspond to overtone and combination bands of functional groups whose vibrational behavior is physically meaningful. Wavelengths around 1200–1300 nm originate from C–H first overtones, those near 1450 nm from O–H stretching overtones, and the region around 1900–1950 nm from O–H combination bands, while N–H combination bands typically appear between 2050 and 2180 nm. Assigning selected wavelengths to these vibrational modes ensures that the resulting models retain chemical interpretability and prevents overfitting driven solely by numerical optimization [63].

In Table S3, the principles, advantages, and limitations of the methods described in the text have been summarized.

Model selection is the first fundamental step in calibration and the models used in NIR spectroscopy can be divided into three broad categories: linear models, nonlinear models, and models based on advanced machine learning. Each is used for different purposes and has specific advantages and limitations.

Linear models assume that the relationship between the spectrum and the properties is a linear combination of the latent variables or components.

They represent the most established and historically used class. Among these, Principal Component Analysis (PCA) is often the starting point for exploring the data matrix. It is an unsupervised technique, as it considers only the internal structure of the spectral data without knowing the analytical objective and thus works on the variables. It is the most well-known and widely used exploratory technique for analyzing data and operates exclusively on the spectral matrix without using any information about the properties to be predicted. The purpose of using this technique is to reduce dimensionality, visualize the data structure, identify anomalous samples and outliers, and evaluate the effectiveness of preprocessing steps.

It practically compresses the spectral matrix X into a combination of orthogonal components, according to the relationship:

X = TP^T^ + E (3)

where T denotes the scores (the representation of the samples in latent space), P contains the loadings (the description of the contribution of the spectral variables to the components) and E represents the residuals.

Therefore, PCA is not a predictive model, but it is important for exploring the structure of the data, identifying outliers, and evaluating the effect of preprocessing [64,65,66].

Conversely, a very simple supervised linear model is the Principal Component Regression (PCR), which combines PCA with linear regression between the scores and the target variable. Since its goal is to predict a numerical value based on NIR spectra, it is a quantitative model. It applies PCA to the spectral matrix X, reducing dimensionality and creating principal components, then uses the PCA scores as predictors in a linear regression against the Y variables [57]. However, since PCR uses PCA scores as predictors, it may incorporate components that are not useful for prediction; therefore, PCR is less efficient and, above all, less widely used than other quantitative models such as Partial Least Squares Regression (PLSR). PLSR is the standard quantitative linear model in NIR spectroscopy, as it constructs latent components that maximize the covariance between the spectral matrix X and the predictive property of Y which represents the quantitative property to be predicted. As in any supervised regression approach, PLSR requires reference values obtained through independent analytical techniques, which provide the calibration targets for the model.

Unlike PCR, PLS incorporates the information contained in Y from the outset.

The PLS model can be expressed as follows [60]:

X = TP^T^ + E (4)

and

Y = UQ^T^ + F (5)

where T and U are the score matrices, P and Q are the loading matrices, and E and F are the residuals. The final relationship between the spectrum and the property is linear:

(6) $$\hat{y}=b_{0}+x\times b$$

where the vector b is obtained by combining weights, loadings, and regression coefficients.

This is the most widely used model because it handles matrices with thousands of highly collinear variables, reduces noise, and uses only the portion of X’s variance that is relevant for predicting Y [67,68].

Multiple Linear Regression (MLR) is the simplest quantitative linear model; however, it is rarely used in NIR spectroscopy because it requires the absence of multicollinearity among the variables, a sample size significantly larger than the number of variables, and a previous selection of wavelengths. Since NIR spectra contain thousands of highly correlated variables, MLR is unstable, overly sensitive to noise, and unable to handle the multivariate structure of the data; therefore, PCR and PLS, as described above, are the preferred operational choices [69,70].

NIR spectroscopy also employs supervised classification models, whose purpose is not to predict a numerical value, but to assign each sample to a discrete class. These models are particularly important, for example, for product authentication, definition of geographical origin, and thus detecting fraud. For this purpose, the most commonly used classification model is Partial Least Squares Discriminant Analysis (PLS-DA), which is derived from PLS, but applied to a response variable that is often encoded in a binary matrix. It constructs latent components that maximize the covariance between the spectral matrix X and the class matrix Y and treats the class as a number. This model can handle extreme multicollinearity, works well even with few samples, is not sensitive to noise, is easy to validate, very stable, and allows samples to be separated into well-defined classes and clusters [71].

Another of the oldest and most widely used models is the Linear Discriminant Analysis (LDA), which assumes that the classes follow Gaussian distributions with identical covariances and that optimal separation between classes is achieved by maximizing the ratio between inter- and intra-class variance [72]. The main gap is that this classification requires the covariance matrix to be invertible, a condition that is rarely met in NIR spectra, where the number of variables generally far exceeds the number of samples, especially in complex matrices such as the food-based ones. Therefore, it is very often replaced by the PLS-DA, which is more robust in the presence of multicollinearity or is applied following dimensionality reduction. In the latter case, we refer to PCA-LDA. This hybrid model combines a PCA decomposition of the spectral matrix with classification using LDA. PCA reduces the dimensionality and removes collinearity, while LDA constructs the discriminant function in the reduced space after selecting the principal components [73,74].

In addition, considering that PCA does not utilize information from Y, it is possible that the resulting separation may be less efficient than that achieved using PLS-DA, which remains the most widely used approach.

A widely used linear classification model is Soft Independent Modeling of Class Analogy (SIMCA), which is based on independent PCA models for each class. Firstly, a PCA model describing the internal variability of each class is constructed. Then, for each new sample, the model evaluates how well it fits each PCA space using two complementary parameters: Hotelling’s T^2^, which indicates how central or extreme the sample is within that space, and the Q-residuals, which quantify the portion of the sample not explained by the PCA model. These values are compared with the confidence intervals and then the sample can be assigned to the class for which both Hotelling’s T^2^ and Q-residuals have a lower value under the threshold [75].

Table S4 summarizes the principal linear model used in NIR spectroscopy.

When the relationship between NIR spectra and their chemical–physical properties can no longer be described by a simple linear combination of variables, non-linear models come into play, introducing more flexible mapping functions [76]:

(7) $$\hat{y}=f(x)$$

where f is a non-linear function defined via a kernel, decision trees, or similarity metrics. In this case, complex curvatures and interactions can be captured, improving predictive capability.

Support Vector Machines (SVMs), particularly in their regression form (SVR), are the most commonly used non-linear models in NIR applications.

Non-linearity is introduced via a kernel, which is a mathematical function that allows the calculation of the similarity between two samples as they had been transformed into a much higher-dimensional space where the separation becomes linear, without actually calculating the transformation.

SVMs are robust and insensitive to noise, making them effective in the presence of moderate non-linear relationships [77].

Another widely applied method involves the use of Random Forest (RF), which is a set of decision trees, each trained on a random subset of samples and variables. The final prediction is the average of the predictions from the individual trees. These models are widely used for very complex datasets which can be well handled without requiring sophisticated preprocessing [78,79].

The k-Nearest Neighbors (k-NN) model is based on local similarity and depends on the geometry of the spectral space. It assigns class labels based on the distance to the k closest samples in the space where k represents the number of neighbors with the smallest distance from the sample according to a chosen metric (typically Euclidean distance), while kernel-PLS is an extension of PLS obtained by introducing a kernel which allows for the capture of non-linear relationships while maintaining the conceptual structure of PLS [80,81,82,83].

Although many non-linear models are available, the most widely used, especially in food analysis, are SVMs.

Table S5 summarizes the non-linear chemometric models discussed in the text, indicating their main applications in classification and quantification.

Machine learning represents an evolution of classical non-linear models and introduces algorithms capable of directly learning complex patterns from the data. Unlike traditional non-linear models, which rely on predefined mathematical transformations, machine learning models autonomously learn the structure of the data through optimization processes.

The most flexible and powerful models used in NIR are Artificial Neural Networks (ANNs), which include Backpropagation Neural Networks (BPNNs), a feed-forward network with one or more hidden layers capable of modeling highly non-linear relationships. However, they require large datasets and rigorous validation.

Radial Basis Function Neural Networks (RBF-NNs) are more stable and simpler than BPNNs; they use radial basis functions as neurons in the hidden layer.

An evolution of classical ANNs is deep learning, which introduces deeper and more specialized architecture. Among these, Convolutional Neural Networks (CNNs), which apply convolutional filters to the spectra, are widely used [84,85,86].

Table S6 reports the principles of the neural-network-based models applied in NIR spectroscopy.

The final goal of any quantitative model is to minimize the difference between predicted and actual values and therefore the model choice depends on the type of data and the desired outcome.

The model’s performance is evaluated using specific parameters such as the mean squared error (MSE) and the Root Mean Square Error (RMSE) which is the most commonly used indicator for this assessment, as it is expressed in the same units used to measure the chemical property [87]:

(8) $$\mathrm{RMSE}\;=\sqrt{\frac{1}{n}}\sum_{i=1}^{n}(y_{i}-{\hat{y}}_{i}{)}^{2}$$

where n is the number of samples, y_i_ is the value obtained for the sample using the reference method, and ${\hat{y}}_{i}$ is the value predicted by the NIR model. The difference (y_i_ − ${\hat{y}}_{i}$) is the prediction error for the sample.

When this error is calculated for the samples used during the model development, it is referred to as Root Mean Square Error of Calibration (RMSEC). A low value for this parameter indicates that the model fits the calibration data well, though it does not guarantee accurate predictions for new samples; therefore, the coefficient of determination (R^2^) is also considered [77,87,88]:

(9) $$R^{2}\;=\;1-\frac{\sum (y_{i}-{\hat{y}}_{i}{)}^{2}}{\sum (y_{i}-\overset{-}{y}{)}^{2}}$$

In this case, $\overset{-}{y}$ is the mean of every y measured sample and this coefficient measures the proportion of variance explained by the model. A value close to 1 indicates a good fit.

Again, to account for the model’s complexity, the Standard Error of Calibration (SEC) is also used. It normalizes the error by the number of estimated parameters, according to the following equation [60]:

(10) $$\mathrm{SEC}\;=\;\sqrt{\frac{\sum (y_{i}-{\hat{y}}_{i}{)}^{2}}{n-k-i}}$$

where k is the number of model parameters. This value is particularly useful in non-linear models, where the risk of overfitting (the model fitting the calibration set too closely) is higher [42].

Table S7 summarizes the main calibration parameters.

### 3.3. Validation

Once the calibration model has been built and trained on a set of samples with known reference values, it is essential to verify its ability to generalize beyond the samples used for training. Therefore, the validation phase is necessary to objectively assess the model’s reliability and predictive robustness.

The validation process can be divided into internal validation, external validation, and advanced validation.

Internal validation is the main method for assessing a model’s ability to generalize. It evaluates the model performance using only samples from the original dataset, estimates the model robustness, identifies overfitting, minimizes the number of latent components, and compares alternative models.

Several strategies can be used, such as Cross-Validation (CV) which is based on the division of the dataset into subsets called folds. These are excluded from the calibration model one at a time and exclusively used as test sets. The process is repeated until all samples have been tested. The parameter used to assess the model’s performance in cross-validation is Root Mean Square Error Cross Validation (RMSECV), which provides a realistic estimate of the predictive error in the absence of an external test.

A more extreme form of cross-validation is the Leave-One-Out (LOO) technique, in which only a single sample is excluded during validation, a prediction is made for it, and the process is then repeated for all samples. This maximizes efficiency even on small datasets; in fact, it is widely used when the number of samples is limited.

A more stable, less noise-sensitive, and less computationally expensive method is k-Fold Cross-Validation, in which the dataset is divided into a fixed number of blocks (*k*) that are typically of similar size, and each block is used in turn as the test set. This is generally the most common form of CV used for datasets of intermediate size [89,90].

When samples are divided into blocks in which samples are selected at regular intervals, the technique is called Venetian Blinds and is used when the samples are ordered to avoid contiguous blocks being too similar. Differently, in Random Subset samples are randomly assigned to the validation blocks [91].

In addition to RMSECV (Equation (11)), other internal validation metrics include the Standard Error of Prediction (SEP) (Equation (12)), which measures the dispersion of prediction errors and is useful for comparing models with different biases (the bias is a parameter that indicates whether the model tends to overestimate or underestimate) (Equation (13)) (Table S8) [49,88].

(11) $$\mathrm{RMSE}\mathrm{cv}=\sqrt{\frac{1}{n}}\sum_{i=1}^{n}(y_{i}-{\hat{y}}_{i,\mathrm{cv}}{)}^{2}$$

(12) $$\mathrm{SEP}=\sqrt{\frac{\sum (y_{i}-{\hat{y}}_{i}{,p)}^{2}}{n-k-i}}$$

(13) $$\mathrm{BIAS}=\frac{1}{n}\sum_{i=2}^{n}(y_{i}-{\hat{y}}_{i}{)}^{2}$$

The most rigorous form of model validation is the external validation, which involves testing the model on an independent set of samples that were not used in any way during calibration (Table S9).

The main parameter used is the Root Mean Square Error Prediction (RMSEP), which represents the model’s actual predictive error under operating conditions. This approach evaluates the model’s actual ability to generalize, identifies any systematic biases, and verifies the model’s stability on new matrices or batches. Other parameters considered in external validation include Residual Predictive Value (RPD) [88]:

(14) $$\mathrm{RPD}=\frac{\;\mathrm{SD}}{\mathrm{RMSEP}}$$

in which SD is the sample standard deviation and assesses how small the predictive error is relative to the actual variability of the dataset. Generally, although RPD values can mathematically exceed 10 or even 50 when the dataset exhibits very high variability, the commonly accepted interpretative scale considers that the model is unusable for values lower than 1.5; differently, if the value is between 1.5 and 2.0 or between 2.0 and 3.0, it can be used for screening processes and approximate quantification, respectively; differently, for higher values it can be considered in the context of a good prediction (a value of 5.0 is considered excellent).

If the dataset is not normally distributed, a very robust parameter is Ratio of Performance to Interquartile Range (RPIQ) [92]:

(15) $$\mathrm{RPIQ}=\;\frac{\mathrm{IQR}}{\mathrm{RMSEP}}$$

in which IQR is the interquartile interval. It is more robust because it does not use the standard deviation but the interquartile interval; therefore, it is particularly useful when there are asymmetric data, outliers, or heterogeneous datasets [49,88].

In addition to internal and external validation, which are the most commonly used methods, there are advanced strategies that allow for a more robust and realistic evaluation of the model, especially with complex datasets (Table S10).

One example is Bootstrap Validation, which generates hundreds of models by repeatedly sampling the dataset with replacement (each sample can be selected multiple times across different bootstrap sets). This procedure allows assessing the stability and variability of the model across multiple resampled datasets. This allows us to estimate the model variability and to evaluate the influence of single observation on the model predictions. This is one of the most powerful strategies when there is a dataset containing few samples [93]. Another strategy used in this type of approach is Monte Carlo Cross-Validation (MCCV), in which the dataset is randomly split into training and test sets multiple times. This allows for the evaluation of the model stability, error variance, and sensitivity to the dataset composition [94]. Differently, in Repeated k-fold, CV k-fold CV is repeated multiple times with different partitions [95].

Block Cross-Validation involves the division of the samples into continuous blocks, thereby preventing samples that are too similar from ending up in either the test or the training sets [96].

Finally, in advanced validation systems, groups of samples are also excluded to assess the model transferability across groups. This strategy is called Segmented CV/Leave-Group-Out [76].

Following the calibration and validation processes, a model is considered robust when RMSEC, RMSECV, and RMSEP have comparable values, BIAS is close to 0, SEP is low, and RPD and RPIQ are higher than 3. If RMSECV is significantly lower than RMSEP, overfitting is present, and the model fails to predict external samples because it has been over-trained on the training test samples [49].

## 4. Application of NIR Spectroscopy in Food Control

In the frame of food safety, NIR spectroscopy is one of the most versatile and strategic techniques for quality control analysis both throughout the production chain and after products enter the market. Today, economic fraud is particularly related to toxicological and allergenic risks and in this context NIR allows for the simultaneous evaluation of multiple chemical and physical characteristics. In fact, it is particularly used for matrices that are extremely different from one another, making it advantageous because traditional techniques often require specific methods for liquid and solid products. Furthermore, it provides results in a very short time (seconds); conversely, the conventional techniques generally require long sample preparation (which can include derivatization and incubation) and time of analysis, and processes that frequently destroy the sample. Furthermore, NIR spectroscopy is a technique that can be used on site, a crucial factor when it is necessary to detect adulteration or contamination as quickly as possible.

### 4.1. Adulteration

The use of NIR in the context of food adulteration is not recent, as it has always been widely employed. However, technological advances in instruments and availability of chemometric techniques have made it even more essential for counteracting illicit commercial practices, which have recently become much more sophisticated, less obvious, and often difficult to detect using traditional methods [97].

NIR spectroscopy has proven highly effective in detecting adulteration in liquid matrices, whose chemical composition can be significantly altered without affecting their organoleptic properties. Considering that NIR signals arise from O–H, C–H, and N–H vibrational overtones, even small compositional changes can be detected, making this technique particularly suitable for identifying fraud in oils, milk, plant-based beverages, and fruit juices. It is evident from the literature that NIR performance depends on four interconnected factors: the chemical detectability of the adulterant, the similarity between adulterant and matrix, the matrix structural characteristics, and the resolution of the instrument combined with the selected chemometric model.

Adulterants that introduce clear spectral perturbations are easier to detect than those that are spectrally similar to the matrix. In palm oil adulteration with lard, Basri et al. (2018) [98] demonstrated that the strong contrast between the unadulterated and adulterated oils enabled excellent performance across instruments of different complexity. FT-NIR and MicroNIR had similar performance (R^2^ > 0.9900, RMSEC ≈ 0.27, RMSEP ≈ 0.34 vs. R^2^ > 0.9900, RMSEC = 0.30, RPSEP = 0.44) after SG and first derivative preprocessing. In fact, LED-MicroNIR, despite its reduced manufacturing complexity, provided robust classification using LDA with high sensitivity (accuracy 93.94% and specificity 0.9333) thanks to targeted wavelength selection (1200 and 1450 nm). A similar situation was observed in the study of Teye et al. (2019) [99], where the presence of Sudan dyes (azo compounds banned for food use due to their carcinogenic potential) produced pronounced spectral signal that enabled portable NIR device to discriminate between authentic and adulterated palm oil samples with identification rates above 95% in both calibration and prediction sets. Likewise, saccharin adulteration in commercial juices generated distinctive NIR features that allowed PLSR models to achieve high sensitivity (R^2^ = 0.9700, RMSECV < 1%) [100]. Taken together, these studies indicated that when adulterants are spectroscopically visible, even low-complexity instruments and linear models can achieve excellent performance.

**Figure 4:**
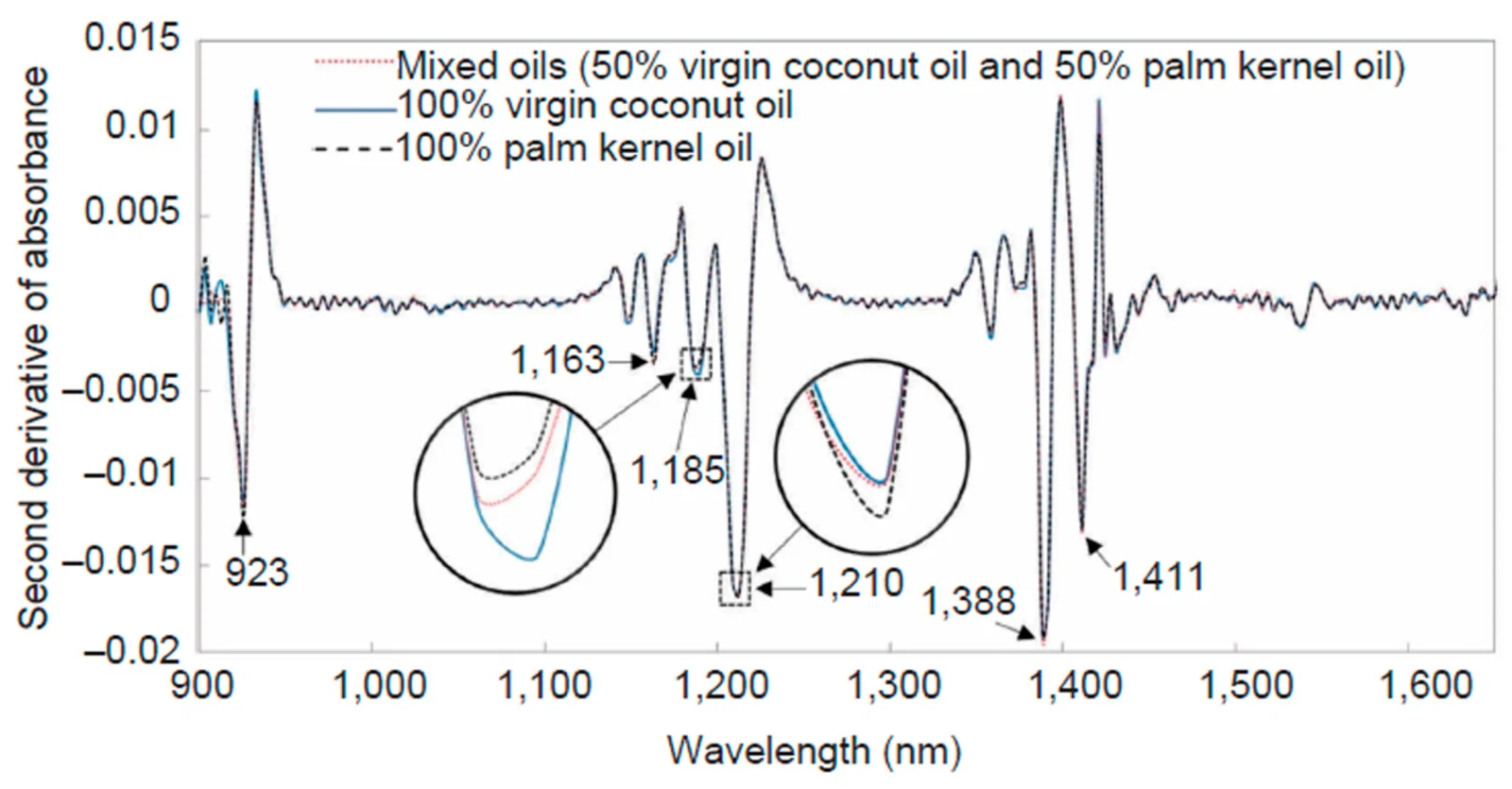
Spectra obtained using the second derivative (2D) method in samples of virgin coconut oil adulterated with palm kernel oil. Blue line: virgin coconut oil; dotted red line: mixed oils (50% virgin coconut oil and 50% palm kernel oil); dotted black line: palm kernel oil (Reproduced from [103]. This article is an open access article distributed under the terms and conditions of the Creative Commons Attribution-NonCommercial-NoDerivatives 4.0 International (CC BY-NC-ND 4.0) license).

A different scenario arises when adulterants share similar vibrational signatures with the matrix. Deng et al. (2024) [101] demonstrated that mineral oil adulteration in corn oil (motivated by economic gain but associated with severe health risks due to the presence of toxic and carcinogenic compounds) required nonlinear models such as SVR, BPNN, and RF to achieve acceptable predictive accuracy (R^2^ ≥ 0.9800, RMSEP < 1.9 mg/kg). This trend was confirmed in peanut oil adulteration with mineral oils, where nonlinear models again outperformed linear ones and provided interpretable classification by identifying the spectral regions most affected by adulteration [102]. Even more challenging is the adulteration of virgin coconut oil (VCO) with palm kernel oil (PKO), a cheaper ingredient with chemical and physical characteristics very similar to VCO. Using hyperspectral imaging (900–1650 nm), a PLS model was developed and achieved R^2^ = 0.9910 and RMSEP = 2.93% with external validation, confirming model robustness (R^2^ = 0.9220, RMSECV = 8.98%) [103]. These studies confirmed that chemically subtle adulterants require higher-resolution instruments and nonlinear models regardless of the food matrix. As reported in Figure 4, the negative peaks at 1185 nm and 1210 nm correspond to second overtones of C–H stretching vibrations. The band at 1185 nm is mainly associated with C–H stretching in methylene groups, and its higher intensity in VCO reflects the greater abundance of medium-chain fatty acids. Conversely, the peak at 1210 nm is linked to C–H stretching modes influenced by unsaturation; its stronger response in PKO is consistent with the higher oleic acid content of this oil.

**Figure 5:**
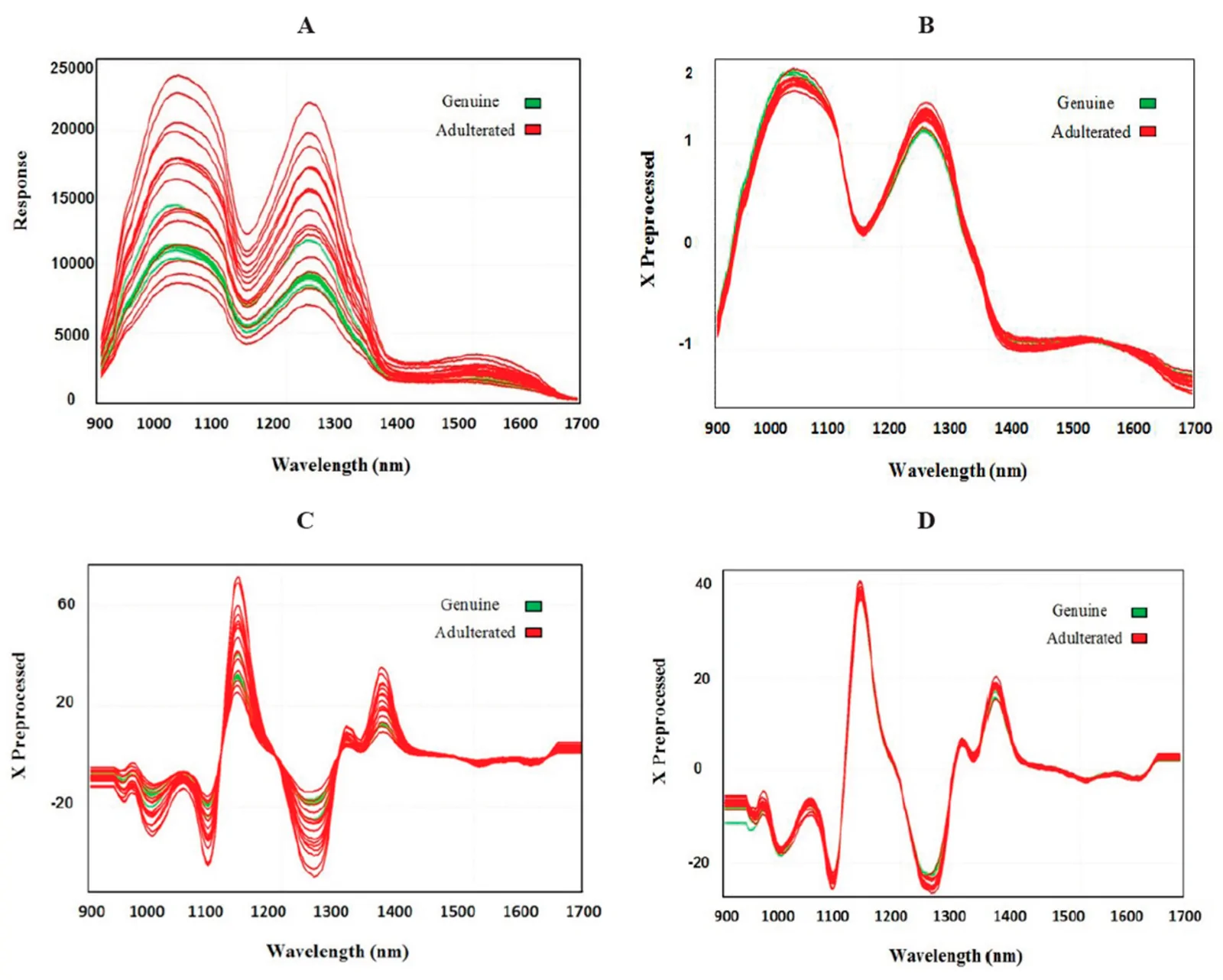
Spectra obtained by analyzing raw data of lime juice samples (**A**) with different processing techniques: SNV (**B**); second-order derivative (**C**), and MSC followed by second-order derivative (**D**) (Reproduced from [108]. This article is an open access article distributed under the terms and conditions of the Creative Commons Attribution-NonCommercial 4.0 International (CC BY-NC 4.0) license).

Matrix composition further influences model behavior. Homogeneous matrices such as cow milk adulterated with water and whey allow variable-selection algorithms and linear models to isolate subtle spectral differences with high precision. Kasemsumran et al. (2007) [104] achieved 100% correct classification using DPLS and SIMCA after MSC and second derivative preprocessing, and PLS quantification yielded RMSEP values of 2.159% (*v*/*v*) and 0.244% (*w*/*v*) for water and whey, respectively. Similarly, goat milk adulteration with cow milk was successfully quantified using miniaturized NIR instrumentation and variable-selection algorithms such as SPA-MLR and iSPA-PLS, with the best model (SGA/iSPA-PLS) achieving R^2^ = 0.9900, RMSEP = 2.15 g/100 g, and RPD = 8.96 [105]. In contrast, emulsified matrices such as coconut milk exhibit natural variability in fat globule size and emulsion stability, which affects model robustness. Water adulteration in coconut milk was easily detected using FT-NIR combined with LDA, SVM, and MLP, achieving 100% classification accuracy [106]. However, adulteration with corn flour or tapioca starch required deep learning approaches such as CNNs to capture more complex and overlapping spectral features, with RPD values as low as 0.20886 [107]. Turbid matrices like lime juice present an additional challenge: scattering effects mask spectral differences and reduce sensitivity. Jahani et al. (2022) [108] demonstrated that adulteration with water and citric acid was more difficult to detect than sugar addition, but PCA, PLS-DA, and kNN combined with SNV and MSC preprocessing enabled discrimination accuracy up to 88% in the external validation set. The above-mentioned studies suggested how matrix homogeneity, emulsion structure, and turbidity directly influence model robustness and preprocessing choice (Figure 5).

Instrumental characteristics also play a decisive role. Benchtop FT-NIR instruments consistently provided the highest precision, as demonstrated in palm oil [98], corn oil [101], milk adulteration with formalin [109], and hyperspectral imaging applied to coconut oil [103]. Formalin (37% formaldehyde solution illegally used as a preservative which poses severe health risks) adulteration up to 50% (*v*/*v*) was successfully detected by combining the use of UV-Vis-NIR spectroscopy with PCA and machine learning models with R^2^ > 0.9980, RMSE 0.16–0.80, and RPD 80.13–182.79 [105]. Benchtop NIR also proved to be highly sensitive in detecting water and sugar adulteration in commercial orange, apple, and grapefruit juices [110]. Conversely, portable instruments excel in rapid screening applications, as demonstrated by Teye et al. (2019) [99] who used handheld NIR devices to discriminate palm oil adulterated with Sudan dyes with high accuracy, and by Liang et al. (2025) [111] who demonstrated that portable NIR can classify milk adulterated with water with high sensitivity and specificity (RMSEP = 0.066% and external validation R^2^ = 0.9760 using SVR). However, when trace levels of adulterants are present or when the matrix exhibits high natural variability, portable instruments clearly decline in performance compared to benchtop systems, which remain the best choice for quantification.

Finally, the choice of chemometric model must be aligned with the complexity of the adulteration scenario. Linear models such as PLS and PLSR perform well when adulterants introduce distinct spectral perturbations, as for palm oil adulteration [98], saccharin detection [103], and several fruit juices [112,113,114]. The evolution of NIR spectroscopy in fruit-juice analysis is clearly visible across three key studies spanning almost three decades. The earliest work, by Twomey et al. (1995) [112], showed that even simple PCA-DA applied to low-resolution NIR data could already distinguish authentic orange juice from samples adulterated with pulp wash, grapefruit juice, or sugar mixtures, reaching 90–95% accuracy. A decade later, Léon et al. (2005) [113] improved the technique by introducing the use of transflectance geometry, which forced light to pass through the sample, reflect on a backing surface, and pass through the matrix a second time. This double-pass configuration increased the effective optical path length and enhanced the visibility of subtle vibrational features, enabling more sensitive quantification of high-fructose corn syrup and sugar mixtures in apple juice, although the sugars’ structural similarity still posed a challenge. The most significant advancement was achieved by Calle et al. (2022) [114], who combined high-resolution NIR instrumentation with modern machine-learning algorithms such as RF, LDA, and SVM. This approach enabled the detection of adulteration in orange, apple, and pineapple juices with grape juice at 97–100% accuracy, and SVR provided quantitative predictions with R^2^ = 0.9890. This evolution further emphasizes the need for new approaches in more complex matrices. Nonlinear models become necessary when spectral overlap increases, as demonstrated in mineral oil adulteration [101,102] and in milk classification tasks [111]. Deep learning approaches such as CNNs offered advantages in highly complex matrices [107], but with reduced interpretability and higher data requirements. Class-based modeling techniques such as DD-SIMCA have proven particularly effective in fruit juice adulteration involving pulp wash and sugar mixtures, achieving high sensitivity and specificity even in challenging scenarios [115]. Overall, the comparison across algorithms suggested that model complexity must increase proportionally to spectral complexity, confirming that no single chemometric approach is universally optimal.

A summary of NIR-based approaches in adulteration detection of liquid food is provided in Table 4.

Solid food products present different challenges compared to liquid matrices, because fraud exploits the physical structure of the product rather than its chemical composition. Flours, spices, coffee, dried fruits, dairy powders, and food supplements can incorporate adulterants without altering appearance, aroma, or texture, making traditional inspection ineffective. In this context, unlike liquid matrices, NIR spectroscopy becomes valuable because solid matrices are heterogeneous, multi-component, and spectrally noisy, requiring chemometric strategies capable of extracting meaningful information from complex signals. A critical evaluation of the literature suggested that solid food frauds fall into four distinct categories—economic, allergenic, toxic, and nutritional/pharmacological—and for each category there are different outputs using NIR spectroscopy.

Economic adulteration is the most intuitive form of fraud, yet its spectroscopic behavior is not always straightforward. In turmeric adulterated with corn starch, the adulterant introduced strong spectral perturbations, enabling FT-NIR combined with PCA and PLSR to achieve high predictive accuracy (R^2^ up to 0.9900, RMSE 0.23–1.3%) and benefiting from targeted band selection via VIP and NAS [116]. When real-world adulteration ranges were simulated (0.1–100%), multimodal Vis-NIR imaging combined with RF models maintained excellent performance (R^2^ = 0.9990, RMSE = 0.391 mg), supported by SNV, MSC, and SG preprocessing [117]. These results might suggest that economic adulteration is always easy to detect, but really it is not always true, as evident in the case of coffee adulteration. In fact, differently from spices, for which adulteration introduced very evident spectral signatures [116,117], Arabica coffee adulterated with Robusta variety or corn generated much subtler spectral variations. Despite the high complexity, micro-NIR models combined with PCA and PLS still achieved high performance (limit of quantification 5–8 wt%, R^2^ > 97%), identifying 16 commercially adulterated samples among 125 blends [118]. A second study confirmed these results for roasted coffee adulterated with corn (1–20%), achieving R^2^ > 0.9600 and RMSEP < 1.5% across the 1100–2500 nm range [119]. The contrast between spices [116,117] and coffee [118,119] reveals a key insight: in solid matrices, the detectability of economic adulteration depends more on matrix heterogeneity and particle distribution than on chemical differences.

A further example comes from lipid-rich matrices, where adulteration produces stronger and more easily interpretable spectral signatures. Clarified butter adulterated with beef tallow—an economic fraud involving cheaper saturated fats—was detected using FT-NIR, with SNV and SG preprocessing enhancing spectral resolution, PCA distinguishing authentic from adulterated samples, and PLSR achieving R^2^ = 0.9500 and RMSEP = 1.53% across the 1–20% range [120]. Compared with powder solid matrices, butter adulteration benefits from intense lipid-associated bands, enabling robust quantification even at low levels. This different behavior between lipid-rich matrices and powder solids underscores how the spectroscopic detectability of economic adulteration strongly depends on the dominant chemical class.

Allergenic adulteration presents a different type of challenge. Garlic powder adulterated with peanut powder demonstrated how NIR can exploit protein-associated C–H and N–H bands to achieve perfect discrimination (100% accuracy, R^2^ = 0.9900, RMSECV = 0.23%) across the 0.5–30% range, using micro-NIR, PCA, and PLS-DA [121]. However, cashew nuts adulterated with peanuts, Brazil nuts, macadamia nuts, or pecans highlighted how botanical similarity can complicate the adulterant detection. Portable NIR achieved near-ideal specificity (≈98%) for all adulterants with the exception of pecans, whose spectral similarity to cashews made them inherently harder to discriminate [122]. These studies [121,122] highlighted that allergenic adulteration is spectroscopically dominated by protein signatures, and that the main limitation was not adulterant concentration, but phylogenetic similarity between adulterant and authentic product.

Toxic adulteration represents the most dangerous category and requires a different spectroscopic strategy. Melamine in infant milk powder is intentionally added to mimic protein content, but it poses severe health risks and is classified as a potential category A2 carcinogen [123]. A non-targeted FT-NIR SIMCA approach achieved an excellent sensitivity and detected melamine, biuret, and sucrose below 1%, thus outperforming portable devices due to higher spectral resolution [124]. Camel milk powder adulterated with urea, ammonium sulfate, starch, and other compounds (2–80%) further demonstrated the importance of one-class classification model such as DD-SIMCA which achieved high sensitivity and specificity (RF reached 98% accuracy, PLSR with R^2^ = 0.9769 and RMSEP = 0.0480) after SNV, MSC, and SG preprocessing [125]. Soybean meal adulteration detection further supported the need for a one-class classification model. In fact, Rodionova et al. [126] used DD-SIMCA to classify samples adulterated with melamine, cyanuric acid, or their mixtures, achieving very good sensitivity and specificity and detecting melamine at 0.07% and cyanuric acid at 0.5%. Both the above-mentioned studies demonstrated that one-class approaches are essential when adulterants are hazardous and present at trace levels, and that solid matrices benefit from anomaly-based detection rather than traditional regression.

Wheat flour adulteration with cassava flour (cassava has a higher glycemic index and poses risks for diabetics, making detection essential for consumers’ health) presents a different challenge. In fact, the complexity did not arise from the spectral similarity between food matrix and adulterant, but from the different spectral patterns registered for the numerous plant varieties of the adulterant. For wheat flour adulterated with eight cassava varieties at five concentration levels (5–40%), multiclass PCA-LDA and PLS-DA models were performed and achieved >90% accuracy for the 2- and 3-class configurations, while the comprehensive 6-class model reached 75.31% [127]. A single generalizable model could also be used for the simultaneous detection of different classes and different concentrations of adulterants—an essential advantage in real-world scenarios where the adulterant is unknown. Although accuracy decreases when classes become too closely spaced, the multiclass approach remains informative and allows the progression of adulteration to be assessed with greater granularity than one class or binary models.

The adulteration of dietary supplements is constantly increasing and represents a significant health risk, since compositional manipulation or the addition of undeclared substances can alter the product’s effectiveness or expose the consumer to undesirable ingredients. In this context, the detection of adulterants requires flexible models capable of handling both the quantification of compositional manipulations and the qualitative classification of added substances. The literature clearly shows this duality, as evidenced in a study in which fish-oil supplements adulterated with vegetable oils or manipulated in their triglyceride/ethyl ester ratios were successfully analyzed using FT-NIR and PLSR, achieving R^2^ > 0.9700 and RMSEP values of 13–16 mg/g for EPA and DHA [128].

A particularly relevant case concerned creatine supplements, which had a non-compliance rate of 21.4% due to dilution with maltodextrin or starch. This non-compliance is not only a regulatory issue but may compromise the product’s effectiveness and pose a risk for consumers who take creatine for sports or clinical purposes. For such matrices, both qualitative models (PLS-DA used to identify the presence of the adulterant) and quantitative models (PLS and MLR used to estimate its level were required, all achieving accuracy above 95% and high RPD values [129].

This phenomenon is even more evident in protein-rich powders, where the adulteration with inexpensive carbohydrate diluents also raises significant safety, nutritional, and regulatory concerns.

While powder carbohydrate-rich matrices often exhibit weak or highly variable spectral perturbations, protein-rich powders behave differently: their amide-related absorption bands provide more structured and informative spectral features, making adulteration easier to detect when carbohydrate-based diluents are introduced. This can be associated with the disruption of the structured amide bands of the protein matrix, generating clear and quantifiable spectral changes. In this context, whey protein concentrate sport supplement adulterated with maltodextrin, flour, or powdered milk was successfully detected using portable NIR and hyperspectral imaging, with PLS-DA achieving 100% accuracy and RMSE values of 0.009–0.026 across the 5–50% range, while PCA provided clear clustering between authentic and adulterated samples [130].

These results illustrate that, unlike heterogeneous carbohydrate powders, protein-rich matrices offer stronger and more coherent spectral signatures, enabling robust quantification even when adulterants are present at relatively low levels.

The above-mentioned literature suggested that adulteration in solid foods is governed not only by chemical adulterant similarity, but also by the matrix heterogeneity, adulterant category, and fraud intent. NIR spectroscopy adapts to these challenges through targeted and non-targeted approaches, multimodal imaging, multiclass models, and one-class algorithms. Its ability to detect economic, allergenic, toxic, and nutritional adulterants across different solid matrices makes it a powerful tool for modern food fraud prevention.

A summary of NIR-based approaches in the detection of adulteration in solid food is provided in Table 5.

### 4.2. Contamination

NIR spectroscopy is a non-destructive technique increasingly used also to detect physical and chemical contaminants in food. Contamination detection is a rapidly expanding and critically important field, as contaminants often pose immediate toxicological risks to consumers. Within this context, mycotoxin detection has emerged as one of the most well-established NIR applications, revealing both the strengths and limitations of NIR-based approaches.

Early work on aflatoxin detection focused on kernel-level discrimination. Using a UV-Vis-NIR system combined with RF, individual kernels contaminated by *Aspergillus flavus* were classified above or below the FDA threshold of 20 ppb, with good sensitivity and specificity of 97% [131]. This study demonstrated that NIR can operate on the single grain, and not necessarily on the bulk sample. In addition, it highlighted a key constraint: performance is strongly dependent on the contrast between contaminated and uncontaminated kernels, which may not always be as pronounced in real storage conditions. Sirisomboon et al. (2013) [132] extended this concept to rice, attempting to quantify both the total percentage of infected grains and the specific presence of yellow-green *Aspergillus*, indicative of potential aflatoxin B1 (AFB1) production. Here, PLSR achieved only moderate performance for total fungi infection (R^2^ = 0.6800, SEP ≈ 29%) and weaker performance for *Aspergillus* infection (R^2^ = 0.4400), despite the presence of clear spectral alterations in regions associated with moisture, starch, and chitin. The analysis of these two studies indicated that kernel-level detection is highly sensitive to matrix variability and infection heterogeneity, and that classical regression models struggle when contamination is non-uniform and spectral signatures are subtle.

A more recent work shifted towards model transferability and deep learning, addressing the need to generalize across matrices and toxins. Deng et al. (2025) [133] proposed a transfer-learning-based approach for detecting AFB1 and Zearalenone (ZEN) in cereals (wheat) and dried fruit (peanuts). Spectral data acquired with FT-NIR and portable spectrometers were transformed into two-dimensional images and analyzed using convolutional neural networks (CNNs), which automatically learn hierarchical spectral features. By reusing pretrained CNNs on new datasets via transfer learning, the study reduced the need for large labeled datasets and achieved R^2^ > 0.9300 for toxin prediction. Compared with the more traditional PLSR approach [132], this work suggested that deep learning and transfer learning can overcome some limitations of matrix variability and limited datasets, but at the same time, model complexity increased and interpretability is reduced, representing an important consideration for regulatory environments.

**Figure 6:**
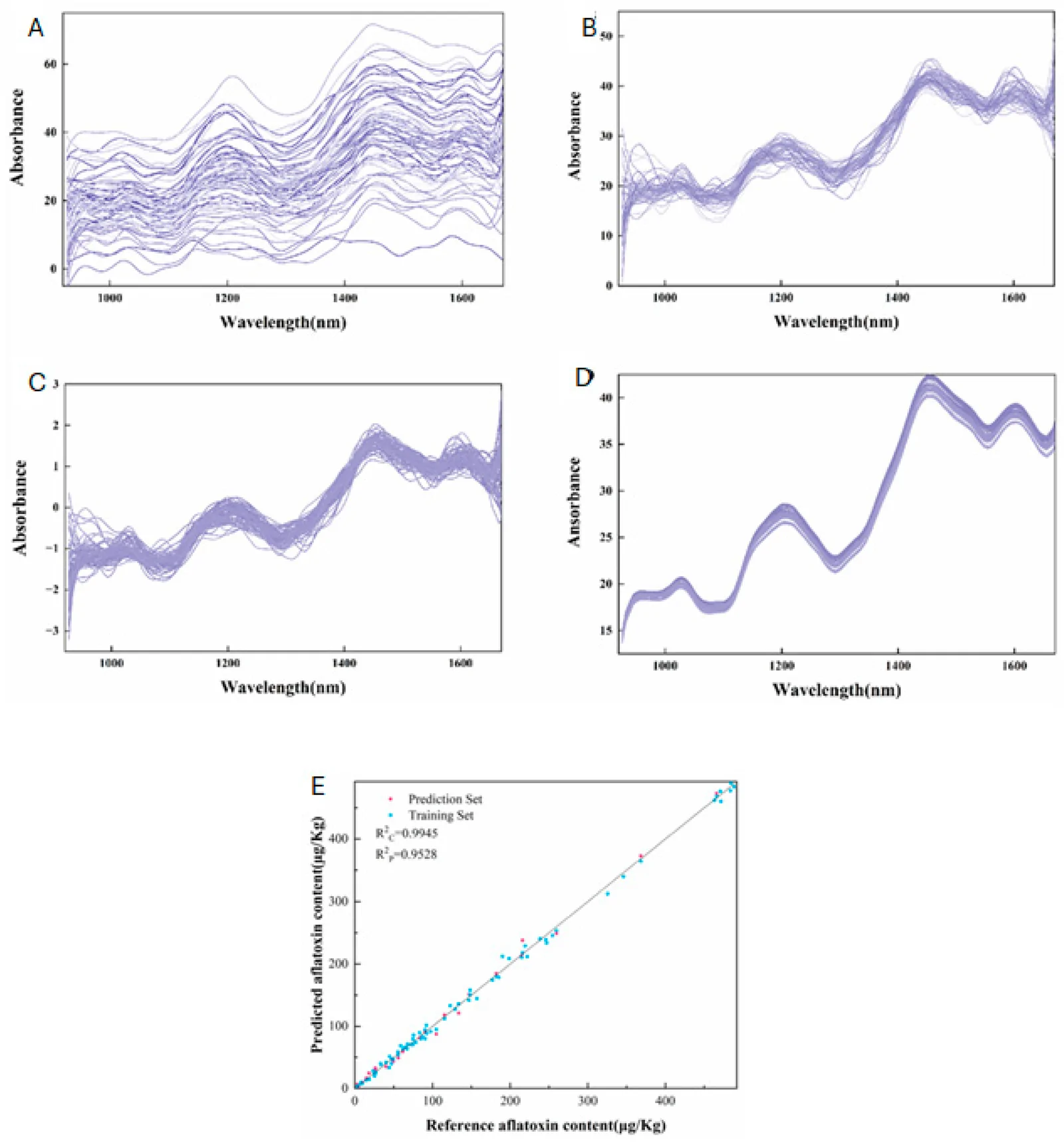
Spectra obtained by analyzing moldy peanut raw data (**A**) and pretreated data using MSC (**B**), SNV (**C**), and SG (**D**). Panel (**E**) represents the SVM prediction model (after SNV preprocessing) for AFB1 content (Adapted from [134]. This article is an open access article distributed under the terms and conditions of the Creative Commons Attribution (CC BY 4.0) license.

Another line of research has focused on dynamic monitoring of fungal growth and toxin accumulation, rather than static classification. In peanuts colonized by *Aspergillus flavus*, NIR spectroscopy was used to track both chemical–physical degradation and AB1 accumulation as a function of temperature and water activity [134]. Preprocessing techniques such as MSC, SNV, and SG were essential to isolate spectral regions sensitive to fungal growth. Nonlinear models (BPNN, RF, SVM) showed particularly high performance, with the SNV-SVM model achieving R^2^ = 0.9945, RMSEC = 9.92, and RPDc = 14.59 in calibration, and R^2^ = 0.9528, RMSEP = 19.58, and RPDp = 7.01 in prediction—values consistent with highly reliable quantitative models. Crucially, spectral changes were observed before measurable aflatoxin accumulation, indicating that NIR can provide early warning of contamination risk. Unlike kernel-level classification [131] and bulk regression [132], this study [134] suggested that time-resolved nonlinear modeling could be more effective for predicting contamination trajectories than static, single-time-point measurements (Figure 6).

In physical contamination, the contaminant is not a chemical species but a foreign body. A 2021 study addressed the detection of iron, plastic, and hair in bread using FT-NIR reflectance combined with artificial vision [135]. Chemometric models based on discriminant analysis and Mahalanobis distance achieved accuracy above 90%, while a modified U-Net CNN enabled automatic localization and segmentation of impurities on bread surfaces, even with irregular shapes and low contrast. Spectral preprocessing (SG smoothing, first and second derivatives) and PCA were used for dimensionality reduction. Compared with toxin detection [131,132,133,134], physical contaminants require integration of spectral and image-based information, and NIR alone is not sufficient without spatial context. In addition, this study represents a broader trend: contamination detection is moving towards multimodal systems that combine spectroscopy, imaging, and advanced pattern recognition.

Allergen contamination, as for gluten in naturally gluten-free matrices, represents another critical area where NIR has to operate at low concentration levels and in complex processing environments. Using a benchtop NIR spectrometer and PLSR, wheat flour additions between 5 and 30% in rice and corn matrices were detected with R^2^ > 0.9400 and RPD values between 4 and 9, both in powder and aqueous suspension samples [136]. In this case, even if the matrix spectrum was not drastically altered by the presence of gluten, the allergen contamination produced a detectable gluten spectral fingerprint, allowing the gluten quantification. Portable devices have further confirmed the possibility to identify the characteristic spectral fingerprint of an allergen, as demonstrated for the wheat flour contaminated with peanut flour. In fact, a contamination level below 2% was detected with accuracy and precision of 0.9937 and 0.9976, respectively [137]. By comparing benchtop [136] and portable [137] system performance, it is evident that instrumental miniaturization does not necessarily compromise performance, provided that appropriate preprocessing and classification strategies are used. However, both the reported studies also underscore a key limitation: NIR detects contamination reliably only when the contaminant sufficiently contributes to the overall spectral signal, which may not be the case for trace-level allergens in highly heterogeneous matrices.

As regards contaminants, all the above-mentioned studies suggested that NIR-based contamination detection is not a single, uniform application but a spectrum of strategies that vary according to contaminant type, matrix, and regulatory context [131,132,133,134,135,136,137]. The critical take-home message is that NIR use is most effective when combined with appropriate model architectures (RF, PLSR, CNN, SVM), tailored preprocessing, and eventually imaging. Moreover, limitations such as matrix variability, uneven contamination, and low-level signals must be explicitly considered when designing food safety monitoring systems.

A summary of the contaminant detection NIR investigations discussed above is reported in Table 6.

### 4.3. Comparison of Analytical Behavior of NIR Instrumentation in Food Control

In the literature reviewed, the differences among NIR instruments are related not only to their analytical capabilities but also to the type of analytical control they impose on the measurement process. Benchtop FT-NIR systems operate as closed, highly controlled environments. They fix the illumination geometry, stabilize the optical conditions, and require consistent sample presentation. Therefore, their calibrations remain stable even when the composition of the raw materials varies, as in the reported case of the detection of mineral oils in edible oils [101,102]. Their strength comes from their ability to suppress procedural variability, but this very high rigidity limits their adaptability when measurements must be performed outside of controlled laboratory environments or when sample preparation cannot be standardized.

Differently, portable NIR devices directly interact with the operating environment. Their measurements depend on environmental conditions, operator handling, and the physical state of the sample at the time of acquisition. The studies of Teye et al. [99] and Liang et al. [111] clearly highlighted how this flexibility enables rapid and decentralized decision-making, but also how small changes in sample orientation or environmental conditions can influence the spectral response. In studies on milk adulteration [104,105], the performance of portable and miniaturized instruments appeared more sensitive to raw-material variability, with calibration models improving only when new batches were incorporated.

HSI systems entirely exhibit different behavior. Rather than stabilizing or adapting to the measurement environment, they reconstruct the spatial organization of the sample. In several studies [103,117], imaging platforms revealed patterns of adulteration that point-based instruments were unable to detect, not because they captured finer spectral details, but because they captured the sample’s structure. Their limitation is the complexity of the data they generate; in fact, spatial information requires advanced algorithms, greater computational resources, and more elaborate workflows, making HSI less practical for routine industrial use even when its analytical capability is superior.

In all these studies, the comparison among the instruments reflects three distinct modes of interaction with the analytical environment: FT-NIR stabilizes it, portable NIR is influenced by it, and hyperspectral imaging reconstructs the entire image of the samples. This perspective suggests that the choice of instrument should be guided by the nature of the measurement environment rather than by spectral performance alone.

## 5. Limitations of NIR Spectroscopy in Food Analysis

Despite its versatility, NIR spectroscopy has several inherent limitations that directly affect its analytical reliability in food control. These limitations emerge from the physical nature of NIR absorption, the architecture of modern instruments, and the constraints imposed by chemometric modeling.

A primary limitation is the nonspecificity of NIR absorption bands. Since NIR signals originate from broad harmonic and combination vibrations, they often represent the convolution of multiple functional groups. This intrinsic spectral overlap reduces selectivity and makes it difficult to isolate the contribution of minor constituents, especially when their vibrational fingerprints match those of the dominant matrix components. This structural characteristic explains why chemically subtle adulterants require increasingly complex models, as observed in the detection of mineral oils [101,102].

A second limitation concerns the instability of the optical–physical interaction. NIR spectra are strongly modulated by scattering phenomena, variations in optical path length, and microstructural differences. These effects are not noise but physical information that interferes with the chemical information. In matrices with variable morphology, such as powders or emulsions, the spectral response becomes a mixture of chemical and physical contributions that cannot be completely separated by preprocessing alone. This structural constraint is independent of the type of adulterant and persists even when using high-resolution instruments [116,117,118,119].

A further constraint is the lack of universal transferability of calibration. NIR instruments can substantially differ in terms of illumination mode, detector type, wavelength range, spectral resolution, and measurement geometry. These architectural differences generate instrument-specific spectral fingerprints that prevent the direct transfer of calibration models. Even when two instruments measure the same sample, the resulting spectra may not be mathematically compatible. This limitation is evident in studies where handheld and benchtop instruments show discrepancies in quantification accuracy [99,103,109,111].

Another limitation is the dependence on model architecture rather than analytical chemistry. NIR is a secondary method, and its performance is determined by the chemometric model, not by the instrument alone. When spectral differences are pronounced, simple linear models suffice; when differences are subtle, model complexity has to be increased. This creates an inherent fragility, meaning that analytical reliability depends on the model’s stability under new conditions. Multiclass adulteration scenarios, such as mixtures of cassava and wheat [127], clearly illustrate how model robustness decreases as the distance between classes narrows, regardless of the instrument’s quality.

A final challenge relates to the difficulty of detecting trace-level analytes. Since NIR does not directly measure analytes but infers them from global spectral models, trace adulterants or contaminants can be masked by the dominant characteristics of the matrix. Single-class classification approaches improve sensitivity [124,125,126], but they require large, representative datasets and are sensitive to variations in raw material composition. This makes it difficult to detect traces under real-world conditions, where variability is high and calibration sets fail to capture all sources of natural variation.

Finally, NIR spectroscopy is limited by the absence of standardized performance metrics. Unlike chromatographic or mass spectrometry platforms, NIR instruments lack universal benchmarks for resolution, signal-to-noise ratio, or repeatability. Therefore, performance depends on the context and cannot be generalized across different matrices or instruments. This inherent limitation complicates regulatory adoption and makes it difficult to compare results across laboratories.

Overall, NIR spectroscopy offers valuable analytical capabilities, but its performance is inherently influenced by spectral nonspecificity, physico-optical interactions, instrument heterogeneity, and model dependence. These limitations must be taken into account when considering its use in food control applications, where reproducibility and analytical accuracy are often critical to protecting public health.

## 6. Future Perspectives

Given the findings presented in this review, NIR spectroscopy is set to become a keystone technique in food control, thanks to instrumental innovation and advanced chemometrics.

Development prospects concern both the technological aspect and the integration of NIR into increasingly automated systems. One of the most promising developments is the miniaturization of devices, which is leading to the widespread adoption of portable sensors that will enable real-time analysis at any point in the supply chain, from the field to distribution.

At the same time, the evolution of data acquisition techniques will enable the simultaneous collection of spatial and chemical information, which could ensure safety and the detection of adulteration and contamination without the need to interrupt the production process.

The integration of this technique with artificial intelligence and machine learning will also enable adaptation to new operating conditions, different instruments, and varying conditions, and this will further allow us to overcome the main NIR spectroscopy limitation consisting of the transfer of models from one instrument to another. All these innovations will be key for expanding the detection of compounds to include even unknown adulterants or contaminants.

Given the minimal use of solvents and the short analysis time, this technology could also be widely adopted in developing countries, so reduction the global food safety gap.

## 7. Conclusions

NIR spectroscopy, especially when combined with chemometric methods and machine learning models, is proving to be a surprisingly practical tool for detecting adulteration and contamination in food. One of its strengths is its simplicity of operation: rapid analysis, no reagents, intact samples. It is an approach that integrates well into routine checks along the agri-food supply chain, without disrupting processes.

The cases analyzed clearly show that NIR can detect even very subtle adulterations, such as the replacement of valuable ingredients with cheaper analogues, even in minimal quantity. The use of supervised models such as PLSR, LDA, and SVM allows us not only to distinguish between authentic and adulterated/contaminated samples but also to estimate the level of adulteration/contamination with good accuracy, which in practice makes a real difference.

Also interesting is the non-targeted approach, which allows us to detect unexpected adulterants/contaminants simply by observing how much a spectrum deviates from the profile of an authentic sample. Meanwhile, the arrival of more flexible models, such as CNNs or transfer learning, is paving the way for systems that are more adaptable to different matrices and multiple adulterations/contaminations, reducing the need for large, labeled datasets.

The integration of NIR spectroscopy with artificial vision systems significantly expands the application of the technique, also allowing the detection of physical contamination localized on the surface of products. In this context, NIR can no longer be considered an emerging technology; in fact, it is already used as an operational tool to address various food safety issues. Its effectiveness stems from the balance between analytical accuracy, process sustainability, and the ability to adapt to different matrices, characteristics that make it compatible with current quality control flows. When predictive models are developed and validated with adequate methodological rigor, the technique’s contribution to fraud prevention, public health protection, and maintaining consumer confidence is concrete and measurable.

## Figures and Tables

**Table 1:** Classification of intentional food fraud.

Fraud Type	Description
Adulteration	Addition of undeclared or unauthorized substances
Substitution	Replacement of high-value ingredients with cheaper ones
Dilution	Addition of liquids to increase volume
Enrichment	Artificial enhancement of sensory/quality traits
Counterfeiting	Illegal reproduction of trademarks or packaging
Document falsification	Manipulation of labels, origin, nutritional claims
Tampering with traceability	Alteration of batches, expiry dates, transport documents
Theft and illegal reintroduction	Re-marketing of seized or stolen products
Unauthorized enhancement	Use of prohibited additives to improve yield or appearance

**Table 2:** Principal techniques used for food fraud detection.

Analytical Technique	Advantages	Limitations	Applicability
ELISA	Suitability for rapid screening	Limited to known targets, risk of false positive	Allergens, mycotoxins, biological residues
LC-MS	High precision, molecular identification	Long processing times, use of non-eco-friendly solvents	Chemical contaminants, residues, adulterants
GC-MS	High sensitivity, volatile compounds analysis	Complex matrix	Flavorings, solvents, organic contaminants
NIR Spectroscopy	Rapid, non-destructive, reagent-free	Complex spectra	Authentication, adulteration, contamination

**Table 3:** Overview of the NIR analytical workflow.

Workflow	Objective	Key Operations	Output
Problem definition	Definition of analytical target and constraints	Selection of matrix, analyte, concentration range, required accuracy	Analytical specifications and model requirements
Measurement geometry selection	Maximization of relevant spectral information	Choice of reflectance, transmittance, or transflectance; set optical path	Optimized optical configuration
Spectral acquisition	Acquisition of stable and reproducible raw spectra	Control of temperature, homogenize sample, set number of scans, average replicates	Raw spectra with metadata
Preprocessing	Reduction in physical variability and noise	SNV, MSC, derivatives (SG), smoothing, detrending	Corrected and normalized spectra
Variable selection	Focus on informative spectral regions	CARS, iPLS, VIP, interval selection, evolutionary algorithms	Reduced spectral space
Modeling	Building of predictive or classification model	PCA (exploration), PLS/PLSR, SVM, ANN	Calibrated model with optimized parameters
Internal validation	Assessment of robustness and avoiding overfitting	Cross-validation, RMSEC, RMSECV, bias analysis	Internal model performance
External validation	Evaluation of generalization capability	Independent test set, RMSEP, RPD	Real-world predictive performance

**Table 4:** Overview of NIR-based approaches for liquid food adulteration detection.

Matrix	Adulterant	Instrument	Model	Preprocessing	Outcome
Palm oil		FT-NIR	PLS-LDA	SNV	High accuracy and low error
	Lard	MicroNIR	PLS	SG + first derivative	High predictive performance
		LED-MicroNIR	LDA	Mean Centering	Strong classification ability
Palm oil	Sudan I-IV	Portable NIR	PCA, SVM, LDA	MSC	High accuracy on-site rapid detection
Corn oil	Other mineral oils	FT-NIR	SVR, BPNN, RF, PLSR	SNV, SG, first derivative	Excellent predictive performance
Peanut oil	Fossil oils	Portable NIR	SVR, RF, BPNN	SNV, SG	Reliable discrimination of adulterated samples
Virgin coconut oil	Palm kernel oil	Hyperspectral NIR imaging	SNV, PLSR	SNV	Excellent predictive ability High sensitivity to low-level adulteration Robust external validation
Cow milk	Water	Portable NIR	SIMCA, NB, kNN, SVM, SVR	SG smoothing, SG derivative, MSC, SNV	High discrimination Excellent predictive ability Robust external validation
Cow milk	Water Whey	FT-NIR	DPLS, SIMCA, PLS	MSC, second derivative	Excellent classification High sensitivity in the quantification of low adulteration levels
Cow milk Buffalo milk	Formalin	UV-Vis-NIR	PCA + ML regressors/classifiers	SG smoothing, MSC, SNV	Outstanding accuracy High predictive power Reliable detection of toxic adulteration
Goat milk	Cow milk	Miniaturized NIR	SPA-MLR iSPA-PLS	SG-smoothing	High accuracy Strong robustness
Coconut milk	Water	FT-NIR	LDA, SVM, MLP, SVR	SNV, MSC, SG smoothing	Excellent classification Reliable prediction in complex lipid emulsions
Coconut milk	Corn flour Tapioca starch	FT-NIR MicroNIR	CNN-based deep learning models	SNV	High predictive performance, High discrimination Robust detection across adulteration levels
Orange juice	Pulp juice, grapefruit juice, sugar mixture	Benchtop NIR	PCA-DA	Raw spectra	High discrimination
Orange juice	HFCS sugar mixture	Benchtop NIR	PLSR, PLS-DA	MSC, SG derivatives	Accurate quantification Strong linearity
Orange juice	Pulp wash Citric acid Sugar	Portable NIR	DD-SIMCA, PLS-DA, RF	SG smoothing, SG derivatives, MSC, SNV	High sensitivity and specificity Robust class-based modeling, Excellent classification of complex multi-component adulteration
Orange, apple, pineapple juices	Grape juice	HR-NIR	RF, LDA, SVM, SVR	SG smoothing, SG first derivative	High accuracy Good linearity Low error
Orange, apple, grapefruit juices	Water Sugars	Benchtop NIR	DA	Raw spectra	Strong discrimination even at low adulteration
Lime juice	Water Citric acid	Handheld NIR	PCA, PLS-DA, kNN	SNV, MSC	Effective detection of acid-based adulteration
Commercial juices	Saccharin	FT-NIR	PLSR	SG, MSC	High sensitivity Reliable detection of adulterant functional groups

**Table 5:** Overview of NIR-based approaches for solid food adulteration detection.

Matrix	Adulterant	Instrument	Model	Preprocessing	Outcome
Turmeric powder	Corn starch	FT-NIR	PCA, PLSR, VIP, NAS	SG smoothing; SNV; SG first derivative	Excellent predictive accuracy, Robust detection
Turmeric powder	Corn starch	Vis-NIR + Multispectral Imaging	RF, SVM, PLS-DA	SNV, MSC, SG smoothing	Outstanding performance
Garlic powder	Peanut powder	NIR sensor	PCA, PLS-DA	Autoscaling, Mean Centering, SG smoothing, SG derivative, SNV	Perfect discrimination High sensitivity to protein-related bands
Roasted Arabica coffee	Robusta coffee corn	MicroNIR	PCA, PLSR	SG	Good linearity Good quantification at low levels
Roasted Arabica coffee	Maize	FT-NIR	PLSR	SG-first derivative	Good linearity Small error in prediction
Milk powder	Melamine, vegetable proteins, inorganic salts, sugars	Benchtop FT-NIR portable NIR	PCA, SIMCA	SG derivatives, SNV, MSC	High sensitivity and specificity for FT-NIR
Camel milk powder	Cow milk powder, goat milk powder, protein powder	Benchtop NIR	PCA, DD-SIMCA, LDA, kNN, RF, PLSR	SNV, MSC, SG derivatives	High sensitivity and specificity for DD-SIMCA High accuracy Low error in prediction Good linearity
Wheat flour	Cassava Flour	MicroNIR	PCA-LDA, PLS-DA	SG smoothing, SG first derivative, SNV	High multiclass discrimination High performance for not extremely close adulteration
Soybean meal	Melamine, cyanuric acid, or mixture	Benchtop NIR	DD-SIMCA	SNV	Excellent sensitivity and specificity
Cashew nuts	Peanuts, Brazil nuts, macadamia, pecans	MicroNIR	SIMCA	SG first derivative, Mean centering	Excellent discrimination
Clarified butter	Beef tallow	Benchtop FT-NIR	PCA, PLS-DA, PLSR	SNV, SG first derivative	Reliable detection of adulteration Clear separation of the adulterated samples
Fish oil supplements	Vegetable oils	FT-NIR	PLSR	SNV, MSC, SG second derivative	Reliable identification and quantification of EPA, DHA, triacylglycerols
Creatin supplements	Maltodextrin, starch	Benchtop NIR	PLS, MLR, PLS-DA	SG	High classification accuracy Strong predictive capability even at low adulteration level
Whey protein concentrate	Maltodextrine, milk powder, flour	Portable NIR and Hyperspectral Imaging	PLS-DA, PCA	SG derivatives, MSC, SNV	High discrimination capacity Clear separation via PCA

**Table 6:** Overview of NIR-based approaches for food matrix contamination detection.

Matrix	Contaminant	Instrument	Model	Preprocessing	Outcome
Peanut	AFB1/ZEN	FT-NIR Portable NIR	CNN Transfer Learning	Smoothing Normalization	High performance Adaptation across matrices and toxins
Peanut	AFB1 risk	Benchtop NIR	SVM, BPNN, RF	MSC, SNV, SG	Strong discrimination Spectral changes detectable before toxin accumulation
Bread	Iron, plastic, hair	FT-NIR artificial vision	DA, Mahalanobis distance, U-Net	SG smoothing, SG derivatives	High accuracy U-Net enables precise contaminant localization
Rice	Aspergillus	Benchtop NIR	PLSR	MSC, SNV, Detrending, SG derivatives	Moderate discrimination Rice variability limits model robustness
Rice	Gluten	Benchtop NIR	PCA, PLSR	Raw spectra	Reliable detection of wheat flour additions Strong predictive ability
Wheat flour	Peanut flour	Portable NIR	LDA	Mean normalization, standardization	High accuracy and precision at very low contamination level
Corn kernel	AFs	UV-VIS NIR	RF	Background subtraction, Mean normalization	High specificity Effective single-kernel classification

## Data Availability

The original contributions presented in this study are included in the article. Further inquiries can be directed to the corresponding author.

## Supplementary Materials

The following supporting information can be downloaded at https://www.mdpi.com/article/10.3390/molecules31173067/s1. Table S1: Features of row pre-treatment used in NIR chemometrics; Table S2: Features of column pre-treatment used in NIR chemometrics; Table S3: Main features of the variable selection methods used in NIR spectroscopy; Table S4: Linear chemometric models used in NIR spectroscopy; Table S5: Non-linear chemometric models used in NIR spectroscopy; Table S6: Machine-learning chemometric models used in NIR spectroscopy; Table S7: Main calibration parameters used in NIR chemometric models; Table S8: Main internal validation parameters used in NIR chemometric models; Table S9: Main external validation parameters used in NIR chemometric models; Table S10: Main advanced systems of validation used in NIR spectroscopy.

## Abbreviations

The following abbreviations are used in this manuscript:

AAC	Administrative Assistance and Cooperation Network
AFB1	Aflatoxin B1
ANN	Artificial Neural Network
AOTF	Acousto-Optic Tunable Filter
BPNN	Backpropagation Neural Network
CARS	Competitive Adaptive Reweighted Sampling
CNN	Convolutional Neural Network
DD-SIMCA	Data-Driven Soft Independent Modeling of Class Analogy
DLP	Digital Light Processing
DMD	Digital Micromirror Device
DSC	Differential Scanning Calorimetry
EFSA	European Food Safety Authority
ELISA	Enzyme-Linked Immunosorbent Assay
EPA	Eicosapentaenoic Acid
FF	Food Fraud Network
FT-NIR	Fourier Transform Near-Infrared Spectroscopy
GA	Genetic Algorithm
GC	Gas Chromatography
GC-MS	Gas Chromatography–Mass Spectrometry
GC-MS/MS	Gas Chromatography–Tandem Mass Spectrometry
HFCS	High-Fructose Corn Syrup
HPLC	High-Performance Liquid Chromatography
HSI	Hyperspectral Imaging
InGaAs	Indium–Gallium–Arsenide
k-NN	k-Nearest Neighbors
LC-MS	Liquid Chromatography–Mass Spectrometry
LC-MS/MS	Liquid Chromatography–Tandem Mass Spectrometry
LDA	Linear Discriminant Analysis
LED	Light Emitting Diode
LTF	Linear Tunable Filter
LVF	Linear Variable Filter
MLR	Multiple Linear Regression
MEMS	Micro-Electro-Mechanical Systems
MIR	Mid-Infrared Spectroscopy
MSC	Multiplicative Scatter Correction
NAS	Net Analyte Signal
NIR	Near-Infrared Spectroscopy
NMR	Nuclear Magnetic Resonance
PbS	Lead Sulfide
PbSe	Lead Selenide
PCA	Principal Component Analysis
PCR	Principal Component Regression
PLS-DA	Partial Least Squares Discriminant Analysis
PLSR	Partial Least Squares Regression
R^2^	Coefficient of Determination
RASFF	Rapid Alert System for Food and Feed
RBFNN	Radial Basis Function Neural Network
RF	Random Forest
RPD	Residual Predictive Deviation
RPIQ	Ratio of Performance to Interquartile Range
RMSE	Root Mean Square Error
RMSEC	Root Mean Square Error of Calibration
RMSECV	Root Mean Square Error of Cross-Validation
RMSEP	Root Mean Square Error of Prediction
SEC	Standard Error of Calibration
SEP	Standard Error of Prediction
SG	Savitzky–Golay
SIMCA	Soft Independent Modeling of Class Analogy
SNV	Standard Normal Variate
SPA	Successive Projections Algorithm
SVM	Support Vector Machine
SVR	Support Vector Regression
UVE	Uninformative Variable Elimination
WPC	Whey Protein Concentrate
ZEN	Zearalenone
