# Supplementary material for: Applications of NIR Spectroscopy and Chemometrics for Food Authentication and Safety: Detection of Adulterants and Contaminants
Source: Molecules. 2026 Aug 31;31(17):3067. doi: 10.3390/molecules31173067 (PMC13566949; doi:10.3390/molecules31173067)
Supplement: Supplementary file 1 [file molecules-31-03067-s001.zip › molecules-4460715-supplementary.pdf]

SUPPLEMENTARY MATERIALS

Applications of NIR Spectroscopy and Chemometrics for Food Authentication and Safety: Detection of Adulterants and Contaminants

Vanessa Pellicorio [1], Raffaella Colombo[1] and Adele Papetti [2,*]

[1] Department of Drug Sciences, University of Pavia, Viale Taramelli 12, 27100, Pavia, Italy
[2] Department of Drug Sciences & C.S.G.I., University of Pavia, Viale Taramelli 12, 27100, Pavia, Italy
* Correspondence: adele.papetti@unipv.it

Table S1. Features of row pre-treatment used in NIR chemometrics.

Row pre-treatment	Purpose	Effect on spectrum
SNV	To normalize spectra and correct multiplicative scatters	Spectra comparable to one other
MSC	To correct spectra for difference in slope and multiplative scatters	Physical variability reduction
Detreding	To remove low frequency trends	Temperature variability and instrumental drift reduction
First derivative SG	To remove constant baseline and highlight broad bands	Baseline offset removing and rapid signal variations highlighting.
Second derivative SG	To remove linear baseline and increase apparent resolution	Hidden bands visible

Table S2. Features of column pre-treatment used in NIR chemometrics.

Column pre-treatment	Purpose	Effect
Mean centering	To bring mean of each variable to zero	Easy construction of models
Autoscaling	To divide each variable by its standard deviation across all samples	Comparable variables
Range scaling	To scale each variable to a defined range	Standardized scale of the variables

Table S3. Main features of the variable selection methods used in NIR spectroscopy.

Method	Principle	Advantages	Disadvantages
VIP	Identification of the most relevant wavelengths	Simple, interpretable	Dependent on PLS structure; not suitable for non-linear relationships
NAS	Adaptative elimination of less informative variables	Flexible; effective for complex patterns	Less interpretable; risk of overfitting
CARS	Elimination of uninformative variables using Monte Carlo sampling and regression coefficients	Produces compact subsets; effective for high dimensional spectra	Instable with small datasets
GA	Use of selection, crossover, and mutation to identify the most informative variables	Very flexible; effective for complex and non-linear patterns	Risk of overfitting
SPA	Selection of variables by minimizing collinearity	Reduces multicollinearity	Does not directly consider model performance
UVE	Evaluation of the stability of regression coefficients and removes variables with low stability	Effective for eliminating noisy or irrelevant wavelengths	May remove relevant variables
iPLS	Splitting spectrum into intervals and building PLS models for each interval	Excellent for localized spectral features; easy to interpret	Interval-dependent; global spectral information are missing; PLS-dependent
biPLS	Splitting spectrum into intervals and iteratively removing the least informative intervals	More robust than iPLS; avoids irrelevant intervals	Interval-dependent; PLS-dependent

Table S4. Linear chemometric models used in NIR spectroscopy.

Model	Type	Principle
PCA	Exploratory	Reduces the dimensionality of the data
PCR	Quantification	Performs PCA on spectra and regresses the response variable on selected principal component
LSR	Quantification	Builds latent variables that maximize covariance between spectra and response variable
MLR	Quantification	Allows a direct linear regression between selected wavelengths and the response variable
PLS-DA	Classification	Uses PLS latent variables to discriminate predefined classes
LDA	Classification	Finds linear combinations of variables that maximize separation among classes
PCA-LDA	Classification	PCA reduces dimensionality; LDA classifies using discriminant functions
SIMCA	Classification	Builds separate PCA model for each class and assigns samples based on distance to class space

Table S5. Non-linear chemometric models used in NIR spectroscopy.

Model	Type	Principle
SVMs	Classification<br>Quantification (SVR)	Uses kernel functions to model non-linear relationships and maximize separation
RF	Classification<br>Quantification	Ensemble of decision trees; predictions based on averaging
k-NN	Classification	Assigns class labels based on the distance from the k closest samples in the feature space
Kernel-PLS	Classification<br>Quantification	Extends PLS by applying kernel function to capture non-linear relationships in latent space

Table S6. Machine-learning chemometric models used in NIR spectroscopy.

Model	Type	Principle
ANNs	Classification<br>Quantification	Learns non-linear relationships through interconnected layers of neurons with adjustable weights
BPPNs	Classification<br>Quantification	ANNs trained by minimizing error through back-propagation of gradients
RBFNNs	Classification<br>Quantification	Uses radial basis activation functions to model non-linear relationships in a localized way
CNNs	Classification	Extracts features through convolutional filters

Table S7. Main calibration parameters used in NIR chemometric models.

Parameter	Principle	Indication	Interpretation
RMSEC	Measurement of the average difference between predicted and reference values	Overall calibration error; model accuracy	Lower RMSEC indicates better calibration performance and smaller deviations.
R^2	Proportion of variance in the reference values explained by the calibration model	Goodness of fit	Values close to 1 indicate strong predictive ability
SEC	Standard deviation of residuals between predicted and reference values	Precision of the calibration model	Lower SEC reflects more stable and precise calibration

Table S8. Main internal validation parameters used in NIR chemometric models.

Parameter	Principle	Indication	Interpretation
RMSECV	Average prediction error obtained during cross-validation	Internal predictive accuracy	Lower RMSEC indicates better internal predictive performance and reduced overfitting
SEP	Standard deviation of prediction residuals for validation samples	Precision of predictions on validation data	Lower SEP reflects more consistent predictions and reduced variability in validation
BIAS	Mean difference between predicted and reference values	Systematic over or under estimation by the model	Positive or negative values reveal systematic drift

Table S9. Main external validation parameters used in NIR chemometric models.

Parameter	Principle	Indication	Interpretation
RMSEP	Measurements of the average prediction error on an independent external dataset	External predictive accuracy and performance on unseen samples	Lower RMSEP indicates strong generalization and reliable prediction capability
RPD	Ratio between the standard deviation reference values and RMSEP	Predictive power relative to sample variability	Higher RPD indicates vetter external prediction
RPIQ	Ratio between the interquartile range of reference values and RMSEP	Robust measure of predective ability	Higher RPIQ reflects strong predictive performance with heterogeneous samples

Table S10. Main advanced systems of validation used in NIR spectroscopy.

Validation Method	Principle	Evaluation
Bootstrap Validation	Repeated resample of the dataset with replacement to generate multiple calibration-validation splits	Model stability
Monte Carlo Cross-Validation	Random split of data into calibration and set of validation many times	Generalization ability under random sampling conditions
Repeated k-fold Cross Validation	Performs k-fold CV multiple times with different random partitions	Predictive performance and model stability
Block Cross-Validation	Samples grouped into blocks and entire blocks left out during validation	Ability to generalize across structured or correlated data
Leave-Group-Out	Data divided into predefined groups, one group is left out at each iteration	Model performance across predefined sample groupings
